# Effect of tillage system on epigeal and foliar insect predation in an organic cropping system in Pennsylvania, USA

**DOI:** 10.1371/journal.pone.0328896

**Published:** 2025-07-31

**Authors:** Shea A. W. Tillotson, Christina A. Voortman, John M. Wallace, Mary E. Barbercheck

**Affiliations:** 1 Department of Entomology, The Pennsylvania State University, University Park, Pennsylvania, United States of America; 2 Department of Plant Science, The Pennsylvania State University, University Park, Pennsylvania, United States of America; Lavras Federal University, BRAZIL

## Abstract

Organic growers rely largely on cultural and biological control to manage pest populations and often use soil disturbance with inversion tillage to manage pests and weeds, incorporate crop residues and fertility amendments, and create seedbeds. Reduced-tillage systems are often associated with greater populations of insect generalist predators, as tillage can directly and indirectly disrupt predators and their activity. We investigated the in-season and legacy effects of soil disturbance in three organic feed grain and one forage production systems that varied in frequency and intensity of disturbance on epigeal predation rates on larval waxworms, *Galleria mellonella* L*.,* and foliar predation rates on eggs of the western bean cutworm, *Striacosta albicosta* Smith, and European corn borer, *Ostrinia nubilalis* (Hübner), by arthropod natural enemies. The experimental site included three annual feed grain production systems comprised of a corn, *Zea mays* L., soybean, *Glycine max* (L.) Merr., and wheat *Triticum aestivum* L. sequence and one forage production system represented by a biculture of alfalfa, *Medicago sativa* L. and orchardgrass, *Dactylis glomerata* L. We also measured damage to corn ears from naturally occurring lepidopteran pests and corn yield. The epigeal predation rate on sentinel waxworms in the system managed predominantly with reduced tillage (64 ± 6.4%) was significantly greater than in the systems managed predominantly with inversion tillage (44 ± 5.5%) or with a shallow high-speed disk (48 ± 5.1%). There was no effect of intensity or frequency of soil disturbance on foliar predation or foliar predator community composition in corn. Damage to corn ears from lepidopteran pests was greater in the system managed predominantly with a shallow high-speed disk (51 ± 4.4% damaged ears) compared to systems predominantly managed with inversion tillage (35 ± 4.3%) or no-till planting (31 ± 1.4%). There was no difference in corn yield across systems, but corn yield variability was greatest in the reduced tillage system. We suggest that the occasional use of inversion tillage with a moldboard plow may not have lasting detrimental effects on foliar or epigeal predation rates on arthropod pests in annual organic grain production systems.

## Introduction

Certified organic crop production systems in the US prescribes a wide range of management practices, including cultural, physical, biological and limited chemical controls to comply with organic regulations [1]. By necessity, organic agronomic crop producers are largely reliant on natural cycles and processes, such as biological control to manage pests, because synthetic insecticides and transgenic varieties are not allowed, and allowed pesticides are often not economical to use [2]. Arthropods contribute to many processes, such as biological control, pollination, decomposition and mineralization, and bioturbation of soil, that contribute positively to agroecosystem health. Agricultural management practices common in organic production, such as crop rotation, use of over-wintering cover crops, maintaining crop residues on the surface, and prohibited use of virtually all synthetic agrichemicals have been associated with lower numbers of insect pests and greater abundance and diversity of predatory arthropods [3–11]. However, organic growers also use practices, such as tillage and cultivation, to manage insect pests and weeds. Frequent or intensive tillage, such as inversion tillage with a moldboard plow, has been associated with negative impacts on soil [4,12–14]. Practices that reduce the frequency or intensity of soil disturbance by tillage in organic systems include adoption of cover-crop based rotational no-till [15], incorporation of perennial phases in the rotation [16], and adoption of non-inversion tillage practices, such as chisel plowing, that may cause relatively lower levels of soil disturbance as compared to an inversion tillage tool, such as the moldboard plow [17]. A tillage tool that has the potential to reduce the level of disturbance relative to inversion tillage with a moldboard plow is the high-speed disk (HSD) [18–20]. The high-speed disk is a shallow non-inversion tillage implement that can be used as a primary tillage tool to incorporate crop residue and control weeds, and to prepare seedbeds following primary tillage. The HSD penetrates soil to a depth of about 10 cm, which is shallower than a moldboard plow, which disturbs soil to a depth of about 15–20 cm. Reducing tillage intensity through the adoption of non-inversion implements or those that operate at a shallower depth could help to retain soil structure and organic matter, while also allowing incorporation of crop and cover crop residues, and fertility amendments; and facilitate weed management and preparation of seedbeds. However, little is known about the effects on predation by arthropods when the HSD is used as a primary tillage and residue management tool or when it is coupled with other conservation practices such as cover crops and crop rotation in organic agronomic crop production systems.

An insect pest of growing importance in the Mid-Atlantic US is the western bean cutworm (WBC), *Striacosta albicosta* Smith (Lepidoptera: Noctuidae). A pest of dry beans and corn, WBC typically emerges from pupae in soil in July, with peak flight and oviposition in August [21,22]. The larval stage feeds in corn ears and can result in economic yield and quality losses [22,23]. Paula-Moraes et al. [23] estimated that one WBC larva per plant can result in a mean loss of 0.945 Mg ha^-1^ corn grain. Through the early 2000’s, distribution of WBC expanded from an endemic range in the western US to the Midwest, Mid-Atlantic, and Northeast US [22] and was first documented in Pennsylvania in 2009 [24]. It has been hypothesized that this range expansion was due to the adoption of reduced- and no-till practices [21,22], as tillage may reduce survival of WBC pupae overwintering in soil. Other hypotheses related to the range expansion of WBC include reductions in pesticide use in agronomic crops, the adoption of *Bacillus thuringiensis* (Bt) transgenic corn hybrids, and climate change [22].

Sentinel prey assays can provide a direct, quantitative measure of arthropod predation under field conditions [25]. Sentinel predation assays with traps baited with last-instar waxworm larvae, *Galleria mellonella* L. (Lepidoptera: Pyralidae), are commonly used to assess epigeal predation rates because of their commercial availability and ease of production, and predatory arthropods may not distinguish their feeding behavior between different pest lepidopteran larvae of comparable size [25–28]. The eggs of the European corn borer (ECB), *Ostrinia nubilalis* (Hübner) (Lepidoptera: Crambidae) are commonly used as sentinel prey for estimating foliar predation rates on pest arthropods, including WBC and other noctuid pests. ECB are easily reared on artificial diet, are commercially available, have a worldwide distribution, and are naturally present during most of the growing season so that predators are accustomed to searching for and finding them [29–31]. Additionally, ECB eggs take four days on average to develop into caterpillars [29], allowing an adequate window of time for observation of predation without risk of larval emergence. For these reasons, ECB can be used as sentinel prey to study foliar predation in the event that natural populations of target pests are rare or absent.

In this study, we investigated the in-season and legacy effects of soil management in an organic, three-year annual feed grain rotation of soybean, *Glycine max* (L.) Merr., winter wheat, *Triticum aestivum* L., and corn, *Zea mays* L., and a perennial forage production system represented by an alfalfa, *Medicago sativa* L. – orchardgrass, *Dactylis glomerata* L. mixture on predation rates on the soil surface and on corn foliage using sentinel prey assays, damage to corn ears caused by lepidopteran larvae, and corn grain yield. We hypothesized that epigeal and foliar predation rates would correspond to the relative levels of soil disturbance among the four systems, with predation rates being lowest in systems with the greatest disturbance, and greatest in systems with the least disturbance; and that corn ear damage and yield loss would be greatest where soil disturbance was greatest, least where soil disturbance was lowest, and intermediate where the HSD was employed as the primary tillage implement.

## Methods

### Site description

This experiment was conducted at the Russell E. Larson Agricultural Research Center in Pennsylvania Furnace, PA (lat: 40°71’88” N, long: 77°96’46” W). This experiment is approximately 4 ha in area, has been managed according to USDA organic regulations since 2011, and received organic certification in 2014 [1]. In 2023, this site was in Zone 6b of the USDA Plant Hardiness Zones [32]. Annually, this site experiences an average of 1,000 mm of precipitation. Mean annual temperatures range from 5˚C to 28˚C. Soils at the site are representative of the Hagerstown Soil Series according to the USDA Natural Resources Conservation Service soil classification system and includes mostly silt loam [33].

### Experimental design

This research was part of a larger multidisciplinary project focused on reducing the frequency and intensity of soil disturbance in organic feed and forage production systems. The four organic cropping systems, or treatments, were designed to produce a gradient in frequency and intensity of disturbance and differed in cover crop species, establishment and terminations method; tillage tool, timing, and frequency of tillage; and in-season crop management (S1 Table). Winter cover crops were grown between each annual cash crop (S1 Table). Systems 1–3 consisted of a three-year, annual crop rotation of corn, *Zea mays* L., Viking O.45-88-P – soybean, *Glycine max* (L.) Merr., Viking O.2155 N – wheat, *Triticum aestivum* L., Malabar in which all crops in the rotation were present in each year (i.e., full entry design), and a fourth system comprised of a biculture of alfalfa, *Medicago sativa* L*.,* King’s 544 PLH and orchardgrass, *Dactylis glomerata* L., King’s Echelon. All seed varieties were USDA certified organic. To maintain the crop rotation in the full entry experiment, each crop entry was initiated with a different crop in the sequence in 2021. For ease, where crop entry is discussed, the abbreviations C-S-W, S-W-C, or W-C-S will be used to indicate the crop sequence, where C denotes corn, S denotes soybean, and W denotes wheat, listed in order of year in which the crop was grown in 2021, 2022, and 2023. The experiment was implemented in a randomized complete block design. Each treatment was replicated four times totaling 48 experimental plots, each measuring 6.1m by 48.7m (S1, S2 Tables).

System 1 used full inversion tillage before corn and soybean and integrated winter cover crops using reduced tillage methods (S1, S2 Tables). Winter wheat was sown in October following the use of a chisel plow to a depth of 15 cm at 202 kg ha^-1^ (S1, S2 Tables). In early March, medium red clover, *Trifolium pratense* L., Albert Lea Seed, VNS was frost-seeded at a rate of 17 kg ha^-1^ into winter wheat using a no-till grain drill. After wheat was harvested in July, the medium red clover was allowed to grow and then mowed to a height of 5 cm in early October. Cereal rye, *Secale cereale* L, Aroostook was then no-till drilled at a seeding rate of 33 kg ha^-1^ in mid-October into established red clover. In late May to early June, corn was planted at 86,000 seed ha^-1^ into a seedbed created with a moldboard plow set to a depth of 20 cm. A cover crop mixture of annual ryegrass, *Lolium perenne* spp. *multiflorum* L.*,* Kodiak, forage radish, *Raphanus sativus* L*.,* Organic Tapmaster, and crimson clover, *Trifolium incarnatum* L*.,* Dixie was interseeded into V4 vegetative growth stage corn after the last cultivation at 28 kg ha^-1^ using a high-clearance, no-till grain drill. The interseeded cover crop mixture was allowed to grow through the following fall and spring, and in late May, soybean was planted at 590,000 seed ha^-1^ into a seedbed created with a moldboard plow set to a depth of 20 cm.

System 2 was managed with shallow non-inversion tillage using a compact high-speed disk (HSD) and was intended to reduce the intensity and depth of soil disturbance before each cash crop. The HSD was set to a working depth of approximately 10 cm. The number of HSD passes necessary to achieve an adequate seedbed differed by crop and year, depending on soil and crop residue conditions. The HSD was used before all cash crop plantings in System 2. Wheat was planted in late October at a seeding rate of 202 kg ha^-1^. After wheat harvest in July, a cover crop mixture of oat, *Avena sativa* L*,* Jerry, forage radish, and Austrian winter pea, *Pisum sativum* Lam*.,* Albert Lea Seed, VNS was drill-seeded at a seeding rate of 22 kg ha^-1^, 1 kg ha^-1^, and 32 kg ha^-1^, respectively. Corn was planted in late May or early June, at a rate of 86,000 seed ha^-1^. After corn harvest, cereal rye was seeded at a rate of 67 kg ha^-1^. Soybeans were planted in late May or early June following high-speed disk tillage at a rate of 590,000 seed/ha^-1^.

System 3 was a reduced tillage system intended to reduce disturbance to the extent possible in an organic grain rotation and used no-till soybean and relay cover cropping practices in wheat. System 3 had the lowest tillage intensity among the annual cropping systems, and the longest period between primary tillage events (S2 Table). In October, wheat was drilled at a seeding rate of 202 kg ha^-1^ following a single pass with the HSD set at 5−10 cm depth. In March, medium red clover was frost drill-seeded into the wheat at a rate of 16 kg ha^-1^. To fully terminate the perennial red clover, a moldboard plow was used before planting corn at a seeding rate of 86,000 seed ha^-1^. This was the only inversion tillage event in the 3-yr crop rotation in System 3. Cereal rye was drilled at a seeding rate of 135 kg ha^-1^ following harvest of corn. The cereal rye was terminated using a roller-crimper in the following spring before no-till planting soybean at a rate of 590,000 seed ha^-1^ into the mat of roll-crimped cereal rye.

System 4 used perennial alfalfa-orchardgrass as a minimal soil disturbance baseline to compare to the three annual cropping systems. In System 4, the alfalfa (29.1 kg ha^-1^) – orchardgrass (8.9 kg ha^-1^) mixture was seeded following wheat harvest and chisel plowing and disking. Because the experiment was full entry, and alfalfa followed wheat in the rotation, the number of alfalfa plots differed by year. In the crop rotation entry that started with corn in 2021, there were four alfalfa plots. In 2022, an additional four plots were planted to alfalfa, and in 2023, four more plots were planted to alfalfa to total 12 alfalfa plots (S2 Table)

### Soil disturbance

We used the number of field operations and a soil disturbance rating (SDR) from the USDA Natural Resources Conservation Service to represent the frequency and intensity of soil disturbance from machinery for each system [34]. The six components of the SDR include soil inversion, soil mixing, soil compaction, soil shattering, soil lifting, and soil aeration. Each component of the SDR is assigned an intensity rating that ranges from 0 (least intense) to 5 (most intense) for each field operation. The total rating for an operation is the sum of the six components. Therefore, SDR ranges from 0, which is the least intense level of disturbance from a field operation, to 30, which is the most intense level of disturbance. The tillage implement with the highest SDR was the moldboard plow, with a rating of 29. The high-speed disk was assigned a rating of 24. No-till planting had an associated SDR value of 5.

The number of all field operations and SDR values for each system were summed starting on January 1 of each year and accumulated through December 31 to provide an annual rating (S3 Table). Annual number of operations and SDR were accumulated across each year from 2021 through 2023 to provide estimates of disturbance across the crop sequence (rotation) in a system. To determine the effects of in-season and rotation number and intensity on predation rates during a year, we accumulated the numbers of disturbances and SDR that occurred before each assessment of predation from January 1 of the sample year and from January 1, 2021, respectively. We included number of days from the most recent field operation as a factor in analyses to determine the effects of the time elapsed since disturbance because we considered that the recency of disturbance could affect observed predation rates.

Multiple consecutive passes using the same equipment were common during the experiment. For example, in System 2 multiple passes with the high-speed disk were sometimes needed to sufficiently incorporate cover crop residue. These multiple passes can account for the greater annual and rotation numbers of disturbances and SDR in System 2 (HSD) than in System 1 (inversion), even though the HSD has a lower SDR than the moldboard plow used in System 1. System 2 was designed to represent an intermediate level of disturbance. Even though management in System 2 sometimes resulted in the greatest overall SDR, we still considered it to represent an intermediate level of disturbance due to the shallow non-inversion action of the HSD. The multiple consecutive uses of the same implement within the same field operation may or may not have similar effects on biological communities as a single use [35]. Therefore, while the number of disturbances and their associated SDR are useful measures for quantifying and comparing the relative levels of disturbance across systems, we acknowledge that there are challenges to its use and interpretation that remain to be addressed through research.

### Epigeal predation assays

To assess relative rates of predation of arthropods on the soil surface, we conducted sentinel prey assays with traps baited with live, last-instar waxworm larvae in all cash crops excluding wheat [8,26–28]. We conducted sentinel predation assays during the field seasons of 2021, 2022, and 2023 following methods described in Rivers et al. [8,9]. Briefly, assay arenas consisting of a cylinder of 19-gauge hardware cloth were used to exclude larger animals [9]. We used waxworm larvae as sentinel prey due to their comparable size to other lepidopteran pests and difficulties in handling and confining pest caterpillars [8,9,26]. We placed two sentinel cages in each treatment plot and placed a sentinel trap consisting of an index card substrate (hereafter, “card”) with five lab-reared waxworms secured with a 1.5 by 2.5 cm length of double-sided hem tape (Aleene’s ©). An opaque white plastic lid was fastened to the top of the assay arenas to exclude rain and the cards and arenas were left in the field for 24 hours, when the sentinel cards and arenas were removed from the field. Any observation of invertebrates on the cards at removal times were recorded. In the laboratory, the waxworm larvae were examined for invertebrate feeding damage to determine predation rates as percentage of larvae with feeding damage or consumed [8,9,26].

### Foliar predation assays

To understand the effect of the three annual crop management systems on predation of arthropod pests on corn foliage, we conducted multiple assays. First, to quantify the effect of the three annual management systems on foliar predation rates, we conducted two types of assays: observational and manipulative egg predation assays. To observe levels of predation on naturally occurring WBC eggs on corn foliage, we conducted a preliminary study in July and August of 2022 in corn in which we observed abundance and distribution of naturally occurring WBC egg masses. The timing of observations was determined based on presence of WBC egg masses on corn plants, and the WBC migration feature on InsectForecast.com [36]. These observations occurred at 1246 (block 1), 1267 (block 3), 1360 (block 4), and 1391 (block 2) WBC growing degree days (GDD), estimated with the NEWA Degree Day Calculator [37]. We used 10˚C as the base temperature, started accumulation of GDD on May 1, and used Rock Springs, Pennsylvania, as the location [38]. Although some research reports suggest March 1 as a start date, a lower threshold of 3.3˚C, and an upper threshold of 23.0˚C for WBC GGD accumulation calculations [39], we used recommendations that growers may more commonly find [40].

To understand the spatial distribution of WBC eggs on corn foliage within plots, we searched leaves on every corn plant in each plot for WBC egg masses in 2022 and applied that understanding to refine sampling in 2023. Each egg mass was visually determined to be WBC by morphological features [22] (S1 Fig). Each egg mass was photographed and labeled with a unique identification number. We made observations over a 7-day period by block, with observations of all corn plots within a block made on the same day. We recorded location of egg masses by plot number, block number, leaf number counted from the bottom leaf, and location of the corn plant within the row within a plot.

In 2023, we conducted a similar assay that included the observation of WBC egg masses and predation to determine relative predation rate on corn foliage (S4 Table). The activity of WBC adults was determined by pheromone trapping and the migration maps feature on InsectForecast.com [36]. We initiated pheromone trapping on June 13 (421 GDD, base 10˚C) and continued until August 28 (1955 GDD), when the number of moths collected in pheromone traps approached zero [38] (S4 Table). We checked pheromone traps twice weekly and collected the first WBC adult on June 26 (627 GDD). Numbers of WBC adults increased beginning on July 7 (866 GDD) and continued through August 4 (1465 GDD), after which, moth numbers decreased dramatically. To collect data on egg predation during peak adult moth flight, we initiated systematic observations of predation on WBC eggs during the week of July 15 (1049 GDD), when WBC adult emergence from overwintering pupae was estimated at less than 25%, and continued through August 11 (1617 GDD), when WBC emergence was estimated at 75% [38,41]. We submitted voucher specimens of adult WBC collected from pheromone traps and eggs collected from corn foliage to the Frost Museum of Entomology at the Pennsylvania State University.

In 2023, we examined 20 plants at five locations within each corn plot for naturally occurring WBC eggs, to total 100 plants per plot. We returned to the same plants over a 6-week period to observe losses due to predation and emergence of larval WBC. Based on preliminary data from 2022, we limited observations to the center 8 rows of each plot, as there was no effect of location of the plant within a plot on presence of WBC eggs in 2022. We flagged, photographed and numbered each egg mass for the purpose of counting and categorizing the fate of individual eggs (predated, hatched, parasitized). At each visit we recorded any new egg masses, and flagged, photographed and assigned a unique number to them.

In 2023, to ensure sufficient egg masses on which to collect predation data and as a comparative method of predator preference, we also conducted sentinel egg predation assays using purchased eggs of the European corn borer, *Ostrinia nubilalis* (Hübner) (ECB, Lepidoptera: Crambidae) (Benzon Research, Carlisle, Pennsylvania). We initiated observation of ECB om 1 May 2023 at 973 WBC GDD (base 10˚C) and ended at 1575 WBC GDD [38]. The ECB eggs were delivered on wax paper and on the same day, were glued to 5 cm x 4 cm green colored index cards with Elmer’s original glue in masses of approximately 50 eggs. The prepared cards were stored at ~4⁰C overnight until they could be deployed in the field on the next morning. Each plot received five prepared cards with ECB eggs. Index cards were stapled to the upper side of corn leaves on plants located adjacent to each of the five locations used for WBC observations. Egg masses were left in the field for a total of 48 hours, with observations at 24 and 48 hours to record predation. Data collected included time of placement, observation, and removal of eggs and/or egg masses, and leaf number on which the index card was stapled, obtained by counting leaves from the soil surface to the observed leaf.

### Foliar predator counts

In 2023, we conducted timed predator counts weekly for six weeks to determine the dominant types of arthropod predators present on corn foliage, starting at 928 GDD (base 10˚C, 1 May 2023) and ending at 1553 WBC GDD. Observations took place during daylight hours from 8am-1 pm, with observations randomized for two blocks occurring on one day, and the remaining two blocks occurring on the next day. The observations were timed to coincide with peak WBC pheromone trap captures and GDD models to assess predators that were present when WBC eggs were available. To scout for predators, we observed 20 corn plants in a row, allowing a 20 second observation period per plant. We identified arthropod predators to the lowest taxonomic level possible with sight identification in the field. Unknown predators were photographed and identified to the lowest possible taxonomic level using keys in Triplehorn et al. [42] and Marshall [43].

### Corn ear damage

We estimated the amount damage on corn ears caused by lepidopteran larvae on August 28, 2023, when most of the corn was in the R5 (dent) stage. We conducted assessments by block at 1955 (blocks 1 and 4), 1974 (block 2), and 1989 (block 3) WBC GDD [38] within the same five locations in each corn plot established for the 2023 egg predation assays. To assess damage, we exposed the corn grain by pulling the husk down to observe the presence of damage and lepidopteran larvae. If a larva was found, we collected it for identification in the laboratory [44,45], and recorded on which plant, block, plot, and system the larva was found. We counted an ear as damaged if any damage related to insects was observed. We submitted voucher specimens of lepidopteran larvae collected from corn ears to the Frost Museum of Entomology at the Pennsylvania State University.

### Yield

Whole corn plots were harvested using a 12-row Case combine with a 9.1m head and grain weight was recorded in the field using a weigh wagon. Two moisture readings were recorded at the time of harvest (John Deere Moisture Chek and Grain Tester SW 16060) and yields were normalized to 15.5% moisture.

### Statistical analysis

All data were analyzed in R [46]. The predation rate on sentinel prey was evaluated using six separate generalized linear mixed effects models with the function “glmmTMB” fitted with a binomial distribution in the package *glmmTMB* [47] and utilizing the function “weights” with the total number of waxworms deployed per observation. Models were evaluated separately due to unequal numbers of alfalfa plots compared to numbers of soybean or corn plots resulting from the full entry design. Each model aimed to ask a specific question about the system.

We used generalized linear mixed effects models to compare relative epigeal predation rates among systems. Because the main tillage treatments (i.e., inversion tillage with a moldboard plow, shallow tillage with a HSD, and reduced tillage with no-till planting) were imposed in soybeans and corn was managed similarly among systems, we determined direct effects of tillage type on soil surface predation rates using data collected from soybean plots. To determine the potential legacy effects of tillage treatments in soybean on predation rates in corn, we ran a generalized linear mixed effects model with data collected in corn plots. Both the soybean and the corn models included system, assay date (early season in June and late season in September), and year (2021–2023) as fixed effects. We used assay date nested within year, within block as random effects to account for non-independence due to repeated sampling of each plot twice per year.

Because of the full entry design, alfalfa plots in each entry varied in age we used data from one- and two-year alfalfa stands to determine the effect of alfalfa stand age on soil surface predation. Fixed effects included age of stand (one- or two-year), assay date (early season in June and last season in September), and year (2022 and 2023). Data for alfalfa in 2021 was not included as there were only one-year alfalfa stands. Assay date nested within year, nested within block were used as random effects to account for repeated observations and block effects.

To compare the effect of cash crop species within a system, we used generalized linear mixed effect models. All models utilized cash crop (corn, soybean, alfalfa), year (2021–2023), assay date (June and September), and an interaction between cash crop and year as fixed effects. Because this full entry experiment was conducted in a corn-soybean-wheat rotation and identity of the crop within a plot changed each year, the interaction between cash crop and year allowed for comparison of cash crop. Assay date nested within year, nested within block was used as the random effect. To compare epigeal predation in corn and soybean managed with inversion tillage (System 1) to alfalfa (System 4), we ran a model that included data from alfalfa, corn managed with inversion tillage, and soybean managed with inversion tillage. To compare epigeal predation rates in corn and soybeans managed with HSD (System 2) to alfalfa (System 4), we ran a model that included data from alfalfa, corn managed with HSD, and soybean managed with HSD. These models compared the perennial forage crop (alfalfa) as a baseline to each of the annual tillage systems. To compare epigeal predation rates in soybean with no-tillage management (System 3), corn managed with no-till in the preceding soybean crop (System 3) to alfalfa (System 4), we ran a model that included data from alfalfa plots, soybean plots managed with no-till practices, and corn plots managed in-season with inversion tillage and with no-till management in the preceding soybean phase of the rotation. This model allowed comparison of the relative importance of current year and legacy effects from management in the previous years in the soy-wheat-corn rotation on soil surface predation.

Significance of fixed effects were evaluated using log-likelihood ratio tests (Wald χ^2^) to compare full versus reduced models using the *anova* function. When models were significant, used the package *emmeans* [48] to compare means with Tukey HSD *post hoc* tests. To determine mean predation rate for each individual grouping (year(s) in stand, cash crop, system), we used the function “group_by” in the package *dplyr* [49].

To analyze the effects of System on foliar predation rates, we used data from WBC egg abundances per plot in 2022 and 2023 summed across all observation dates. Data from WBC egg predation rates from 2023 were calculated as means of observed predation on individual egg masses. For generalized linear mixed-effects models, we used the function “glmer” from the package *lme4* [50]*.* For WBC egg abundances in 2022 and 2023, we used a generalized linear mixed effects model with a negative binomial distribution. Both abundance models used block as a random factor and system as a fixed effect.

A generalized linear mixed effects model with a negative binomial distribution was used to fit a model of WBC egg predation rates in 2023. Fixed effects included system with block as a random factor. Models were evaluated using a log-likelihood ratio test and the Wald χ^2^ test statistic [51]. The function “anova” was used to determine the significance of main fixed effects and their interactions.

We used an orthogonal contrast *post hoc* test to conduct a planned comparison of in-season management approaches in corn: Systems 1 and 3 (moldboard plow) vs System 2 (high-speed disk). This *post hoc* test used the function “contrast” to weight Systems 1, 2, and 3, as −0.5, 1, and-0.5, respectively. To calculate the predation rates in each system, we summed the number of predated eggs observed in each system and divided by the total number of WBC eggs in that system. To calculate the mean predation rate of the entire field, we summed the number of predated eggs observed and divided by total number of eggs observed.

We used a generalized linear mixed effects model with a binomial distribution to fit a model of presence or absence of predation on individual WBC egg masses. This was to allow comparison between WBC predation and ECB predation as the binomial model provided the best fit for the ECB egg predation data. Egg masses were assigned “0” for no predation and “1” for any amount of predation. System was the fixed factor and block was the random factor. We used an orthogonal contrast *post hoc* test to conduct a planned comparison of in-season management approach. To determine the effect of system on ECB egg predation rates in corn in 2023, we used the function “glmer” to model presence or absence of predation. System was the fixed factor and block and assay date were random factors. We used the function “anova” to determine the significance of System. We assigned any egg mass that experienced any amount of predation a “1” and any egg mass that did not experience predation a “0”. We used a *post hoc* test with the function “contrast” to weight systems in a planned comparison of Systems 1 and 3 vs System 2, coded as −0.5 (System 1), 1 (System 2), and −0.5 (System 3), respectively.

To analyze the predator community compositions of each system, we conducted a permanova from the PERMANOVA package [52]. To compare the insect predator community composition of each system, we used non-metric dimensional scaling (NMDS) to visually represent the dissimilarity of predator communities with *ggpubr, ggvegan, and ggrepel* [53–55]. Using the “ggplot” function from the package *ggplot2* [56], we overlaid the NMDS values with polygon shapes representing the 95% confidence interval for each system using *vegan* [57]. For both the NMDS plot and the PERMANOVA, only predators that were observed in more than 1% of all observations were included. To compare predator abundance between systems, we used a generalized mixed model with a negative binomial distribution within the package *MASS* [58] using the function “glmer.nb”. System was the fixed factor and block was the random factor. Total abundance of all predators observed was included for the comparison of predator abundance among systems.

To compare the effects of system on caterpillar abundance on or in corn ears, we used a generalized linear mixed effects model with a Poisson distribution with fixed effect system and random effect block. When the model was significant, we used an orthogonal contrast *post hoc* test to compare the effects of the HSD with the moldboard plow. To compare the effects of system on ear damage, we used a linear model with function “lm” and ran the model into an ANOVA with the function “aov” including system as a fixed effect and block as a random effect. When the model was significant, we used an orthogonal contrast *post hoc* test to compare the in-season tillage management approaches: moldboard plow vs high-speed disk. An ANOVA combined with an orthogonal contrast *post hoc* was also used to compare the effect of system on yields.

## Results

### Epigeal predation assays

**Fig 1:**
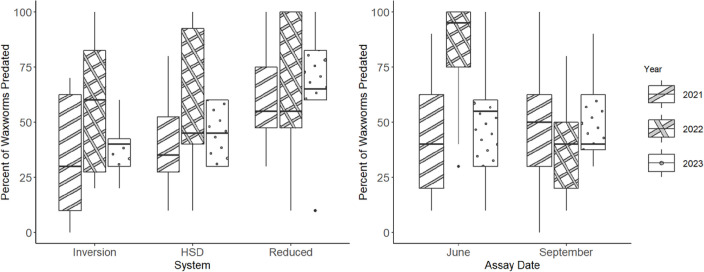
Percent of waxworm larvae predated in Systems 1 (inversion), 2 (HSD), and 3 (reduced tillage) in soybean in 2021, 2022, and 2023 (left panel). Percent of waxworm larvae predated on assays conducted in June and September in 2021, 2022, and 2023 (right panel). HSD = High-speed disk.

There was a significant effect of system on epigeal predation of sentinel waxworms in soybean (χ^2^ = 24.8, df = 2, p < 0.01), but no effect of assay date (χ^2^ = 3.2, df = 1, p = 0.07) or year (χ^2^ = 2.8, df = 2, p = 0.25). Epigeal predation rates in System 3 (reduced tillage) were significantly greater than in System 1 (inversion tillage) (p < 0.01) or System 2 (HSD) (p < 0.01). Mean epigeal predation rates were 43.8 ± 5.5%, 47.9 ± 5.1%, and 63.8 ± 6.4% in soybean in Systems 1, 2, and 3, respectively. The interaction of year and assay date was significant (χ ^**2**^ = 18.0, p = 0.06) (Fig 1). Epigeal predation rates in June were significantly lower in 2021 than in 2022 (p < 0.01). All other comparisons were not significant.

**Fig 2:**
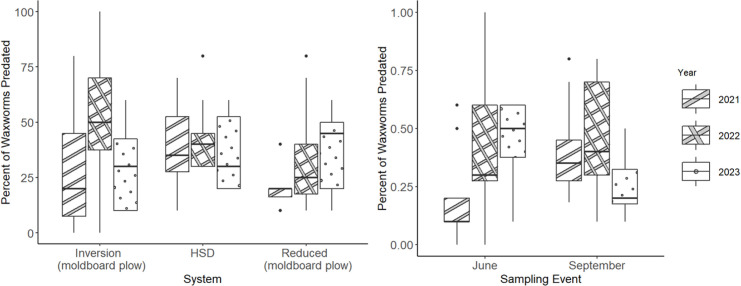
Percent of waxworm larvae predated in corn in Systems 1 (inversion), 2 (HSD), and 3 (reduced tillage in soybean followed by inversion tillage in corn) (left) and by year and assay event (right). HSD = High-speed disk.

There were no significant effects of system (χ^2^ = 5.1, df = 2, p = 0.08), year (χ^2^ = 4.3, df = 2, p = 0.11), or assay date (χ^2^ = 0.2, df = 1, p = 0.70) on epigeal predation rates in corn (Fig 2). Mean epigeal predation rate on sentinel larvae in corn across systems was 35.5 ± 2.6%.

There was no significant effect of alfalfa stand age (1 vs 2-yr; χ^2^ = 3.5, df = 1, p = 0.06), assay date (χ^2^ = 0.1, df = 1, p = 0.77), or year (χ^2^ = 0.1, df = 1, p = 0.82) on epigeal predation rates in alfalfa. The mean epigeal predation rate on sentinel larvae in alfalfa was 48 ± 6%.

When comparing the effects of corn and soybean managed with inversion tillage (System 1) to alfalfa (System 4) on epigeal predation rates, there was a significant main effect of cash crop (χ^2^ = 7.7, df = 2, p = 0.02), but not assay date or year. Epigeal predation rates in System 4 (alfalfa) were greater than in corn (p = 0.02). There was a significant interaction between crop and year (χ^2^ = 14.7, p < 0.01). Mean epigeal predation rates on sentinel larvae were 47.8 ± 4.8%, 36.7 ± 5.7%, and 43.8 ± 5.5% in alfalfa, corn, and soybean, respectively.

When comparing predation rates in soybean and corn in System 2 (HSD) to alfalfa (System 4), there was no difference in epigeal predation rates due to crop (χ^2^ = 5.1, df = 2, p = 0.08), year (χ^2^ = 0.1, df = 2, p = 0.67), or assay event (χ^2^ = 0.1, df = 1, p = 0.73). Mean predation rates on sentinel *G. mellonella* were 47.8 ± 4.8%, 39.6 ± 4.4%, and 47.9 ± 5.1% in alfalfa, corn, and soybean, respectively.

When comparing epigeal predation rates in soybeans in System 3 (no-till planting), and corn in System 3 (managed with reduced-tillage in the preceding soybean crop and inversion tillage in-season), there was a significant effect of cash crop (χ^2^ = 58.5, df = 2, p = < 0.01) on epigeal predation rates, but no effect of year (χ^2^ = 0.2, df = 2, p = 0.90) or assay date (χ^2^ = 0.1, df = 1, p = 0.74). Epigeal predation rates were greater in soybean than in alfalfa (p < 0.01) and corn (p < 0.01). Mean predation rates were 47.8 ± 4.8%, 30.3 ± 4.1%, and 63.8 ± 5.6% in alfalfa, corn, and soybean, respectively.

### Foliar predation assays

#### Western bean cutworm egg abundance and predation.

In 2022, we observed 12,329 WBC eggs in a total of 204 egg masses, based on observation of every corn plant in each plot. There was no statistically significant difference in egg abundance among systems (χ^2^ = 1.1, df = 2, p = 0.60). There were 3,306 eggs observed in System 1 (26.8 ± 2.5% of all eggs observed); 4,685 in System 2 (37.9 ± 1.7% of all eggs observed); and 4,338 in System 3 (35.3 ± 1.8% of all eggs observed).

In 2023, we observed 7,984 WBC eggs in a total of 116 egg masses, based on observation of 100 corn plants in each plot. There was no statistically significant difference in egg abundance among systems (χ^2 ^= 0.12, df = 2, p = 0.94). Using an orthogonal contrast *post hoc* test, there was no statistically significant difference in egg abundance between in-season management approaches when comparing systems with in-season inversion tillage (Systems 1 and 3) and System 2 (HSD) (p = 0.76). The mean percentage of corn plants with at least one WBC egg mass was 8.75%, 10.5%, and 7% in Systems 1, 2, and 3, respectively.

In 2023, there was no difference in WBC egg predation rates by system (χ^2 ^= 1.7, df = 3, p = 0.64). When comparing presence and absence of predation on WBC egg masses on corn among the three annual cropping systems, there was no difference in number of egg masses experiencing any amount of predation (χ^2^ = 0.86, df = 2, p = 0.65) (S5 Table). When comparing in-season management approaches, there was no difference in the number of WBC egg masses experiencing predation among systems (p = 0.58). The mean predation rate of WBC egg masses experiencing any level of predation on corn was 30.2 ± 3.9%. When comparing in-season management approaches, there was no difference in WBC egg predation between systems employing the moldboard plow (Systems 1 and 3) and systems employing the HSD (System 2). The entire field experienced a mean predation rate per WBC egg mass of 13.5%. In 2023, the field experienced a mean hatch rate per egg mass of 68.8%. Parasitism was observed in 1.6% of all WBC egg masses and was not considered further.

### European corn borer egg predation

In 2023, we observed 33 deployed sentinel ECB egg masses comprising a total of 4,851 eggs. There was no difference in number of ECB egg masses experiencing any amount of predation among systems (χ^2^ = 0.81, df = 2, p = 0.67) or in-season tillage management (p = 0.64). Deployed egg masses experienced a mean predation rate of 12.9 ± 1.8%. When including only egg masses that experienced any predation, 77.7 ± 4.6% of ECB eggs were predated per egg mass.

### Timed predator counts

We observed 2,315 predatory arthropods on corn foliage over a six-week period. The most common taxa observed were Anthocoridae (66% of all observations), Coccinellidae (19% of all observations), Araneae (6% of all observations), and parasitoid wasps (4% of all observations). Predator community composition (F_2,59 _= 1.5, p = 0.23) (S2 Fig) and predator abundance (χ^2^ = 0.57, df = 2, p = 0.75) were similar across systems and in-season tillage management (p = 0.62) (S2 Fig).

### Caterpillar abundance and corn ear damage

During a 3-day period, we observed 329 WBC caterpillars on corn plants. Caterpillar abundance in System 2 was significantly greater than in System 1 (p < 0.01) or System 3 (p < 0.01). Caterpillar abundances were 96 (29.2 ± 2.1% of all caterpillars observed), 145 (44.1 ± 10.2%), and 88 (26.7 ± 3.2%) in Systems 1, 2, and 3, respectively. When comparing in-season management approaches, WBC abundances in systems using inversion tillage (Systems 1 and 3) were significantly lower than where the HSD (System 2) was used (p < 0.01). Of the 390 caterpillars collected from corn ears, the majority (329 caterpillars, 84%) were identified as WBC.

We observed greater ear damage in System 2 (51 ± 4.4%) from lepidopteran pests than in System 1 (35 ± 4.3%) or in System 3 (31 ± 1.4%) (F_2,12 _= 7.7, p = 0.02). In planned comparisons by tillage implement (moldboard plow vs high-speed disk), systems employing the moldboard plow (Systems 1 and 3) suffered lower rates of ear damage compared with System 2 which employed the HSD (p < 0.01).

### Corn yield

There was no difference in corn grain yield among systems (F_2,12 _= 2.80, p = 0.14). In 2023, mean corn yields were 10.12 Mg ha^-1^, 8.26 Mg ha^-1^, and 9.10 Mg ha^-1^ in Systems 1, 2, and 3, respectively. There were no differences in corn grain yield in planned comparisons of in-season management approaches using the moldboard plow (9.61 ± 0.5 Mg ha^-1^) and the high-speed disk (8.26 ± 0.4 Mg ha^-1^) (p = 0.09). Mean yield for the entire field was 9.2 ± 0.4 Mg ha^-1^. The coefficient of variation was 4.29, 9.50, and 23.8 for Systems 1, 2, and 3, respectively.

## Discussion

We investigated the effects of organic crop management systems varying in frequency and intensity of soil disturbance on epigeal predation rate on sentinel waxworms and the foliar predation rate on naturally occurring western bean cutworm eggs and sentinel European corn borer eggs as indicators of the effects of management systems on the potential for natural control of insect pests. We expected tillage frequency and intensity in the current and previous year in the rotation to affect predation rates on the soil surface and on corn foliage. We also investigated the predatory arthropod community composition on corn foliage, caterpillar damage to corn ears, and corn yield in Systems 1–3.

### Tillage effects on epigeal predation

We expected that tillage frequency and intensity in the current and previous year of the rotation to affect predation rates on the soil surface and on corn foliage. We expected that epigeal predation rates would be, in order from least to greatest, in System 1 (inversion tillage) which experienced the greatest intensity of tillage, System 2 (HSD), System 3 (reduced tillage), and with the greatest epigeal predation in System 4 (perennial alfalfa – orchardgrass). We observed that tillage intensity affected epigeal predation on sentinel prey in the soybean phase of the rotation, with the lowest intensity of tillage resulting in the greatest epigeal predation rates. Greater epigeal predation rates in System 3 could be due to a greater abundance of epigeal predators, which would be consistent with a meta-analysis that found greater abundance of epigeal predators in reduced tillage systems compared to systems that included any intensity of tillage [10]. Although we did not assess activity-density of epigeal predators in our study, the authors of the meta-analysis suggest that predators that spend any part of their life cycle in the soil can potentially be disrupted by tillage, even at shallow depths [10]. Greater predator abundance could potentially contribute to greater epigeal predation rates [59].

### Crop effects on epigeal predation

The annual rotation in our study included corn, soybean, and wheat. A perennial forage system was included to provide a contrast to the greater levels of soil disturbance in the annual cropping systems. We hypothesized that there would be similar rates of epigeal predation in the corn and soybean phases of the rotation within the same system, as they experienced similar levels of soil disturbance. We found that reducing in-season soil disturbance had a greater effect on epigeal predation rates than the legacy of soil management or crop in the preceding year. When comparing predation in the corn and soybean phases within System 1 (inversion tillage) to System 4 (alfalfa), epigeal predation rates were lower in corn than in alfalfa and similar to those in soybean. Although soil disturbance ratings and predator abundance were not directly correlated in our study or in Rowen et al. [10], arthropods can be directly or indirectly negatively affected by soil disturbance [60,61]. The abundance or activity of predatory arthropods can be directly affected by tillage or indirectly by reducing potential shelter through disruption of surface residues and reduction of prey in the soil [8,10,62–65]. Corn and soybean experienced similar soil disturbance ratings, and we suggest this directly affected epigeal predation rates.

In both soybean and corn, the interaction between assay date and year significantly affected epigeal predation rates. Predation rates generally increase over the summer season, especially in reduced tillage cropping systems [66,67]. Variations in precipitation, temperature, and other weather conditions can greatly influence predator abundance and activity [68]. The weather conditions early in the growing season may influence predation rates later in the season, and annual variation in weather could explain the significance of the interaction between year and assay date that we observed in our experiment.

The greater epigeal predation rates in soybean compared to alfalfa was an unexpected result. Predation is affected by multiple factors including the effects of the crop environment on predator abundance, community composition and activity. We suggest that even though alfalfa experienced fewer soil disturbances than soybean, factors other than soil disturbance contributed to the observed predation rates on sentinel prey. For example, alfalfa may have hosted higher populations of predators and prey organisms which could have distracted from predation on sentinel prey through predation on preferred non-sentinel prey or intraguild predation [69,70]. Additionally, the greater density of alfalfa stems compared with the lower density of soybean stems may have impeded movement of predators and could possibly have resulted in lower predation rates on sentinel prey [71,72].

Previous studies have shown that the greater predator abundance and activity in forages, including alfalfa, are associated with greater levels of pest suppression by natural enemies compared with annual cropping systems [7]. Low levels of soil disturbance and stability of a system can conserve predatory arthropods [73]. Denys and Tscharntke [73] found significantly greater predator:prey ratios in 6-year-old perennial field margins compared with 1-year-old perennial field margins. We hypothesized that age of alfalfa stand (one- vs. two-year) would affect epigeal predation rates with two-year alfalfa resulting in greater predation rates than in one-year stands. However, we did not observe a difference in predation rates due to age of alfalfa stand in the short-term. It is possible that differences could develop over longer time frames.

### Foliar predation rates

We expected that oviposition by WBC would be similar across Systems 1–3, but that systems with reduced tillage would have greater predator abundance and/or diversity, which would result in greater predation and lower damage to corn ears and greater yield compared to systems with greater frequency and intensity of tillage. Although a study conducted in 1990 observed greater oviposition by ECB in corn managed with a chisel plow compared to ridge-tillage or no-tillage [74], we found no effect of tillage management on oviposition by WBC. Additionally, there was no effect of tillage intensity on foliar predation rates on corn foliage in 2022 or 2023. These findings could be due to the relatively small plot sizes and the mobility of WBC and natural enemies throughout the field. In-season management practices had a greater effect on epigeal predation than the legacy of previous management. Management with inversion tillage resulted in similar epigeal and foliar predation rates regardless of how the systems were managed in the previous year. Specifically, we observed similar epigeal predation and foliar predation rates in the corn phase of the rotation in Systems 1 (managed in both the previous soybean and corn crops with inversion tillage) and 3 (managed in previous soybean crop with no-tillage and in-season with inversion tillage in corn).

The pattern of egg predation varied somewhat between naturally occurring WBC egg masses and sentinel ECB egg masses. We observed that when a WBC egg mass experienced predation, a mean of ~30% (16 eggs) of the eggs in the egg mass were predated. In contrast, if an ECB egg mass had any predation, a mean of ~77% (13 eggs) of the eggs within the egg mass experienced predation. WBC egg masses can contain anywhere from 2–345 individual eggs per mass, with a mean of 85 eggs per egg mass [22], whereas ECB masses typically include 15–30 individual eggs per mass [75]. The greater number of eggs in WBC egg masses than in ECB egg masses could explain our observed differences in egg predation rates in which ECB egg masses were more commonly entirely consumed compared to WBC egg masses, even though similar numbers of eggs were consumed regardless of species (S5 Table).

Multiple economic thresholds for WBC have been proposed, including 4%, 8%, and 20% of plants with WBC egg masses [23]. Local recommendations for commercial non-organic growers suggest insecticide application when WBC egg masses are observed on 5% of plants [76]. With an average of 9% of plants with at least one egg mass, our experimental plots reached an economic threshold based on local recommendations. We detected a mean WBC hatch rate per individual egg mass of 69%. This hatch rate is below that observed for non-organic systems, which has been reported at a 97% hatch rate [21], indicating a level of natural control greater in this organic system than has been reported for conventional systems [6,77–79].

The community composition and abundance of foliar predators were similar across management systems, despite our expectation to observe differences. This could be due to the plot size, as predators observed on corn foliage, especially winged predators, are able to move freely throughout the field. Of predators observed, three of the most common four taxonomic groups (Coccinellidae, Anthocoridae, hymenopteran parasitoids) have adult life stages with wings, making movement throughout site possible [80–81]. Predators have been observed to travel between highly managed and natural areas, annual and perennial crops, cash crops and cover crops, field margins and inner-fields, and between cash crops within the same field [7,8,62,66,69,81–83]. The plot size used in this experiment would have allowed insect predators to disperse throughout the field and may explain similar predator community composition and abundance across systems.

Although a similar abundance of naturally occurring WBC eggs and similar levels of predation occurred across systems, System 2 (in-season and legacy of management using the HSD) experienced the greatest level of damage to corn ears by lepidopteran pests, predominantly from WBC. It is possible that slight variations in WBC egg abundance across systems resulted in greater differences in ear damage as each WBC larva can cause significant damage. One WBC egg mass has been observed to cause damage and infestations in an area of 1.8 to 3.1 meters in diameter [21]. Although there were no statistical differences in abundance of WBC eggs among systems, a small difference may have produced the significant difference in damage to corn ears from caterpillars that we observed among systems.

### In-season management effects

When comparing the effects of in-season management practices, we detected a greater similarity between System 1 and System 3 (with moldboard plow) compared to System 2 (HSD). Systems 1 and 3 had similar WBC egg abundances, caterpillar abundances, corn damage, and yield. Although a soil disturbance rating was not observed to directly impact predatory arthropods in our study or in a meta-analysis [10], others have shown that arthropods can be negatively affected by disturbance [60–61]. Abundance, diversity and activity of arthropod natural enemies can be affected by tillage directly by causing mortality, and indirectly, through reducing prey and shelter from surface residues [8,10,62–65]. We suggest that the similar soil disturbance ratings within System 1 (240) and System 3 (291) contributed to the similar predation rates that we observed in those systems (S5 Table). However, when comparing in-season management in corn, the greater intensity rating (SDR) for the HSD (SDR = 330) compared to the systems with inversion tillage (SDR = 240), could have contributed to differences observed between System 2 and Systems 1 or 3, even if differences in foliar predator communities and predation rates were not detected.

We observed similar corn yields in System 1, managed in both corn and soybean with inversion tillage, and System 3, with a legacy of no-till management in previous phases of the rotation. Although we expected corn yield to be related to tillage intensity and resulting levels of damage by WBC to corn ears, corn yield was statistically similar across management systems. Reducing tillage in organic cropping systems does not *per se* negatively affect profitability of organic cropping systems [14]. There are many factors that contribute to and influence yield, including insect pest pressure, weed competition, weather patterns and soil legacy effects, such as soil compaction, water holding capacity, and fertility levels [13,15,19,84–86]. Even so, it is important to acknowledge that even relatively small differences in yield can affect profitability. According to 2024 USDA organic feed corn prices, the average organic farm in Pennsylvania (56.25 hectares) [87–88], could lose about $150,000 when experiencing a yield loss of 0.95 Mg ha^-1^ [88]. Our findings differ from previous studies on tillage and legacy of soil management practices. In a study published in 2015, cover crop most strongly differentiated soil arthropod communities, but after three years of treatment, tillage intensity was a predictor for soil arthropod communities [89]. That earlier study may indicate that the legacy of tillage intensity on soil arthropod communities may develop over a longer time frame. However, the greater impacts of in-season management and the lack of legacy effects of tillage that we observed suggest that the occasional use of inversion tillage may not have lasting negative effects on epigeal predation rates. We also observed the greatest variability in corn yield in System 3, which reduced the amount of soil disturbance relative to Systems 1 and 2. This variability in grain yield could lead to unstable farm income and difficultly planning and managing farm finances [90]. We suggest that growers interested in reducing disturbance to soil may consider a tillage rotation that includes both tilled and no-till phases, to maintain arthropod predator communities and reduce the potential risk of yield losses due to variability during the no-till phase.

### Conclusion

Organic growers rely on natural processes and cycles, such as natural control of insect pests by arthropod natural enemies to keep arthropod pest populations below economically damaging levels. Epigeal, but not foliar, predation rates by arthropod natural enemies were negatively related to in-season, but not legacy, soil disturbance from tillage. The use of no-till planting soybeans resulted in a greater level of epigeal predation compared to the moldboard plow or HSD. Although the high-speed disk may be considered less disturbing to soil as an occasional management tool, when used as a primary tillage tool, it was associated with unexpected negative effects on corn ear damage from caterpillar pests. For growers seeking to improve soil health and encourage epigeal arthropod predation, incorporating no-till practices where appropriate and practicable, may be a better solution than reliance on the high-speed disk as a soil management tool. We suggest that in the short-term, in-season management has a greater effect than the legacy of management in the preceding crop. This is promising for growers who are concerned about using inversion tillage in their rotation because the occasional use of inversion tillage may not necessarily have lasting detrimental effects on beneficial soil organisms and potential for natural control of pests by arthropod natural enemies.

## Supporting information

S1 Table Management strategies for four organic feed grain and forage systems showing the cropping sequence for Entry 3 (Wheat-Corn-Soybean) (after Tillotson et al. 2025). (DOCX)

S2 Table Field operations by experimental system, rotation entry point, and date (after Tillotson et al. 2025). (DOCX)

S3 Table Annual and accumulated frequency and estimated soil disturbance ratings (SDR) for experimental rotation entries and cropping systems. (DOCX)

S4 Table Schedule of activities for predation on eggs of Western bean cutworm (WBC) and European corn borer (ECB) on corn foliage in 2023. GDD = Growing Degree Days, Rock Springs, PA, using the NEWS Cornell GDD calculator (https://newa.cornell.edu/degree-day-calculator/).
(DOCX)

S5 Table Total number of western bean cutworm (WBC) egg masses, total number of individual WBC eggs observed, and mean percent of hatched and predated WBC egg mass observed in corn in 2023. (DOCX)

S1 Fig Freshly oviposited western bean cutworm egg mass on corn leaf. Morphological features include white coloration, round dome shape, and “dumpling-like” indents on dorsal side.
(DOCX)

S2 Fig NMDS plot depicting arthropod predator community composition on corn foliage in 2023. Taxonomic groups that accounted for greater than 1% of all observations including: COCC (Coccinellidae), ANTH (Anthocoridae), ARAE (Aranae), PARA (parasitoid wasps). The green shape represents the inversion tillage system (moldboard plow), the orange shape represents the shallow tillage system (high-speed disk), and the purple shape represented the reduced tillage system (moldboard plow in corn, no-till planting in the previous soybean crop).
(DOCX)

## References

[1] Part 205 – National Organic Program. National Archives Code of Federal Regulations. 2000. https://www.ecfr.gov/current/title-7/subtitle-B/chapter-I/subchapter-M/part-205

[2] Durham TC, Mizik T. Comparative economics of conventional, organic, and alternative agricultural production systems. Economies. 2021;9(2):64. doi: 10.3390/economies9020064

[3] Birkhofer K, Bezemer TM, Bloem J, Bonkowski M, Christensen S, Dubois D, et al. Long-term organic farming fosters below and aboveground biota: Implications for soil quality, biological control and productivity. Soil Biology and Biochemistry. 2008;40(9):2297–308. doi: 10.1016/j.soilbio.2008.05.007

[4] Headrick D. The future of organic insect pest management: be a better entomologist or pay for someone who is. Insects. 2021;12(2):140. doi: 10.3390/insects12020140 33562223 PMC7914490

[5] Kremen C, Iles A, Bacon C. Diversified farming systems: an agroecological, systems-based alternative to modern industrial agriculture. Ecol Soc. 2012;17:44. doi: 10.5751/es-05103-170444

[6] Mäder P, Fliessbach A, Dubois D, Gunst L, Fried P, Niggli U. Soil fertility and biodiversity in organic farming. Science. 2002;296(5573):1694–7. doi: 10.1126/science.1071148 12040197

[7] Prasifka JR, Schmidt NP, Kohler KA, O’neal ME, Hellmich RL, Singer JW. Effects of living mulches on predator abundance and sentinel prey in a corn–soybean–forage rotation. en. 2006;35(5):1423–31. doi: 10.1603/0046-225x(2006)35[1423:eolmop]2.0.co;2

[8] Rivers AN, Mullen CA, Barbercheck ME. Cover crop species and management influence predatory arthropods and predation in an organically managed, reduced-tillage cropping system. Environ Entomol. 2018;47(2):340–55. doi: 10.1093/ee/nvx149 29471320

[9] Rivers A, Voortman C, Barbercheck M. Cover crops support arthropod predator activity with variable effects on crop damage during transition to organic management. Biological Control. 2020;151:104377. doi: 10.1016/j.biocontrol.2020.104377

[10] Rowen EK, Regan KH, Barbercheck ME, Tooker JF. Is tillage beneficial or detrimental for insect and slug management? A meta-analysis. Agriculture, Ecosystems & Environment. 2020;294:106849. doi: 10.1016/j.agee.2020.106849

[11] Tuck SL, Winqvist C, Mota F, Ahnström J, Turnbull LA, Bengtsson J. Land-use intensity and the effects of organic farming on biodiversity: a hierarchical meta-analysis. J Appl Ecol. 2014;51(3):746–55. doi: 10.1111/1365-2664.12219 25653457 PMC4299503

[12] Cavigelli MA, Mirsky SB, Teasdale JR, Spargo JT, Doran J. Organic grain cropping systems to enhance ecosystem services. Renew Agric Food Syst. 2013;28(2):145–59. doi: 10.1017/s1742170512000439

[13] Oldfield EE, Bradford MA, Wood SA. Global meta-analysis of the relationship between soil organic matter and crop yields. SOIL. 2019;5(1):15–32. doi: 10.5194/soil-5-15-2019

[14] Pearsons KA, Chase C, Omondi EC, Zinati G, Smith A, Rui Y. Reducing tillage does not affect the long-term profitability of organic or conventional field crop systems. Front Sustain Food Syst. 2023;6. doi: 10.3389/fsufs.2022.1004256

[15] Wallace JM, Barbercheck ME, Curran W, Keene CL, Mirsky SB, Ryan M, et al. Cover crop–based, rotational no‐till management tactics influence crop performance in organic transition within the Mid‐Atlantic United States. Agronomy Journal. 2021;113(6):5335–47. doi: 10.1002/agj2.20822

[16] González del Portillo D, Arroyo B, Morales MB. The adequacy of alfalfa crops as an agri-environmental scheme: A review of agronomic benefits and effects on biodiversity. Journal for Nature Conservation. 2022;69:126253. doi: 10.1016/j.jnc.2022.126253

[17] Cooray A, Rejesus RM, Aglasan S, Li Z, Woodley A. The impact of conservation tillage intensities on mean yields and yield risk. Soil Security. 2023;12:100096. doi: 10.1016/j.soisec.2023.100096

[18] Abbott LK, Manning DAC. Soil health and related ecosystem services in organic agriculture. SAR. 2015;4(3):116. doi: 10.5539/sar.v4n3p116

[19] Cooper J, Baranski M, Stewart G, Nobel-de Lange M, Bàrberi P, Fließbach A, et al. Shallow non-inversion tillage in organic farming maintains crop yields and increases soil C stocks: a meta-analysis. Agron Sustain Dev. 2016;36(1). doi: 10.1007/s13593-016-0354-1

[20] Watts CW, Patterson DE. The development and assessment of high speed shallow cultivation equipment for autumn cereals. J Agricultural Engineering Research. 1984;29(2):115–22. doi: 10.1016/0021-8634(84)90065-9

[21] Hagen AF. The biology and control of the western bean cutworm in Dent Corn in Nebraska1. J Economic Entomology. 1962;55(5):628–31. doi: 10.1093/jee/55.5.628

[22] Michel AP, Krupke CH, Baute TS, Difonzo CD. Ecology and management of the western bean cutworm (Lepidoptera: Noctuidae) in Corn and Dry Beans. J Integrated Pest Management. 2010;1(1):A1–10. doi: 10.1603/ipm10003

[23] Paula-Moraes S, Hunt TE, Wright RJ, Hein GL, Blankenship EE. Western bean cutworm survival and the development of economic injury levels and economic thresholds in field corn. J Econ Entomol. 2013;106(3):1274–85. doi: 10.1603/ec12436 23865192

[24] Tooker JF, Fleischer SJ. First Report of Western Bean Cutworm (Striacosta albicosta ) in Pennsylvania. Crop Management. 2010;9(1):1–4. doi: 10.1094/cm-2010-0616-01-rs

[25] Lövei GL, Ferrante M. A review of the sentinel prey method as a way of quantifying invertebrate predation under field conditions. Insect Sci. 2017;24(4):528–42. doi: 10.1111/1744-7917.12405 27686246

[26] Birkhofer K, Bylund H, Dalin P, Ferlian O, Gagic V, Hambäck PA, et al. Methods to identify the prey of invertebrate predators in terrestrial field studies. Ecol Evol. 2017;7(6):1942–53. doi: 10.1002/ece3.2791 28331601 PMC5355183

[27] Grieshop MJ, Werling B, Buehrer K, Perrone J, Isaacs R, Landis D. Big brother is watching: studying insect predation in the age of digital surveillance. American Entomologist. 2012;58(3):172–82. doi: 10.1093/ae/58.3.172

[28] Lundgren JG, Shaw JT, Zaborski ER, Eastman CE. The influence of organic transition systems on beneficial ground-dwelling arthropods and predation of insects and weed seeds. Renew Agric Food Syst. 2006;21(4):227–37. doi: 10.1079/raf2006152

[29] Kim KC, McPheron BA. Evolution of insect pests. New York, NY: John Wiley & Sons. 1993.

[30] Nagy B. Rearing of the European corn borer (*Ostrinia nubilalis* Hbn.) on a simplified artificial diet. Acta Phytopath Acad Sci Hung. 1970;5:73–9.

[31] Siegfried BD, Hellmich RL. Understanding successful resistance management: the European corn borer and Bt corn in the United States. GM Crops Food. 2012;3(3):184–93. doi: 10.4161/gmcr.20715 22688691

[32] United States Department of Agriculture. Plant Hardiness Zones. Available from: https://planthardiness.ars.usda.gov/. 2023.

[33] Soil Survey Staff. Web Soil Survey. Available from: https://websoilsurvey.nrcs.usda.gov/app/. 2019.

[34] NRCS. Natural Resources Conservation Service - Revised Universal Soil Loss Equation, Version 2 (RULES2). United States Department of Agriculture. 2008. Available from: https://www.nrcs.usda.gov/conservation-basics/conservation-by-state/maryland/revised-universal-soil-loss-equation-version-2

[35] Kemanian AR, Stöckle CO. C-Farm: A simple model to evaluate the carbon balance of soil profiles. European J Agronomy. 2010;32(1):22–9. doi: 10.1016/j.eja.2009.08.003

[36] InsectForecast. Available from: https://www.insectforecast.com/.

[37] NEWA Degree Day Calculator. Available from: https://newa.cornell.edu/degree-day-calculator/.

[38] DeGaetano A, Moore R, Belcher B, Eck B. CSF growing degree day calculator. Available from: http://climatesmartfarming.org/tools/csf-growing-degree-day-calculator/. 2016.

[39] Hanson AA, Moon RD, Wright RJ, Hunt TE, Hutchison WD. Degree-day prediction models for the flight phenology of western bean cutworm (Lepidoptera: Noctuidae) assessed with the concordance correlation coefficient. J Econ Entomol. 2015;108(4):1728–38. doi: 10.1093/jee/tov110 26470314

[40] NEWA Western Bean Cutworm Flight Forecast. Available from: https://newa.cornell.edu/western-bean-cutworm/.

[41] Smith JL, Difonzo CD, Baute TS, Michel AP, Krupke CH. Ecology and Management of the Western Bean Cutworm (Lepidoptera: Noctuidae) in corn and dry beans—revision with focus on the great lakes region. J Integrated Pest Management. 2019;10(1). doi: 10.1093/jipm/pmz025

[42] Triplehorn CA, Johnson NF, Borror D, DeLong DM. Borror and DeLong’s Introduction to the Study of Insects. 7 ed. Independence, KY, USA: Brooks/Cole, Cengage Learning. 2023.

[43] Marshall S. Insects: their natural history and diversity: with a photographic guide to insects of eastern North America. 2nd ed. Richmond Hill, ON, Canada: Firefly Books Ltd. 2017.

[44] Eaton AT. Identifying common caterpillars in sweetcorn. Available from: https://extension.unh.edu/sites/default/files/migrated_unmanaged_files/Resource002121_Rep3132.pdf.

[45] Munkvold GP, White DG. Compendium of Corn Diseases. 4th ed. St. Paul, MN, USA: APS Press. 2016.

[46] R Core Team. A language and environment for statistical computing. Available from: https://www.r-project.org/. 2022.

[47] Brooks M E, Kristensen K, Benthem K J ,van, Magnusson A, Berg C W, Nielsen A, et al. glmmTMB balances speed and flexibility among packages for zero-inflated generalized linear mixed modeling. The R Journal. 2017;9(2):378. doi: 10.32614/rj-2017-066

[48] Lenth RV. Emmeans: estimated marginal means, aka least-squares means. https://rvlenth.github.io/emmeans/.

[49] Wickham H, Francois R, Henry L, Muller K, Vaughan D. dplyr: a grammar of data manipulation. Available from: https://dplyr.tidyverse.org/.

[50] Bates D, Mächler M, Bolker B, Walker S. Fitting linear mixed-effects models Usinglme4. J Stat Soft. 2015;67(1). doi: 10.18637/jss.v067.i01

[51] Bolker BM, Brooks ME, Clark CJ, Geange SW, Poulsen JR, Stevens MHH, et al. Generalized linear mixed models: a practical guide for ecology and evolution. Trends Ecol Evol. 2009;24(3):127–35. doi: 10.1016/j.tree.2008.10.008 19185386

[52] Vicente-Gonzalez L, Vicente-Villardon JL. PERMANOVA: Multivariate analysis of variance based on distances and permutations. Available from: https://cran.r-project.org/web/packages/PERMANOVA/index.html.

[53] Kassambara A. ggpubr: “ggplot2” based publication ready plots. Available from: https://rpkgs.datanovia.com/ggpubr/.

[54] Simpson GL, Oksanen J. ggvegan: “ggplot2” plots for the “vegan” package. Available from: https://github.com/gavinsimpson/ggvegan. 2023.

[55] Slowikowski K. Ggrepel: automatically position non-overlapping text labels with “ggplot2.”. Available from: https://ggrepel.slowkow.com/. 2023.

[56] Wickham H. ggplot2: elegant graphics for data analysis. 3rd ed. New York: Springer-Verlag. 2016.

[57] Oksanen J, Simpson GL, Blanchet FG, Kindt R, Legendre P, Minch PR. vegan: community ecology package. Available from: https://github.com/vegandevs/vegan. 2022.

[58] Venables WN, Ripley BD. Modern Applied Statistics with S. 4 ed. New York: Springer. 2002.

[59] Jacobsen SK, Sigsgaard L, Johansen AB, Thorup-Kristensen K, Jensen PM. The impact of reduced tillage and distance to field margin on predator functional diversity. J Insect Conserv. 2022;26(3):491–501. doi: 10.1007/s10841-022-00370-x

[60] Rusch A, Bommarco R, Ekbom B. Conservation biological control in agricultural landscapes. Advances in Botanical Research. Elsevier. 2017: 333–60. doi: 10.1016/bs.abr.2016.11.001

[61] Stinner BR, House GJ. Arthropods and other invertebrates in conservation-tillage agriculture. Annu Rev Entomol. 1990;35(1):299–318. doi: 10.1146/annurev.en.35.010190.001503

[62] Almdal CD, Costamagna AC. Annual crops contribute more predators than perennial habitats during an aphid outbreak. Insects. 2023;14(7):624. doi: 10.3390/insects14070624 37504631 PMC10380491

[63] Henneron L, Bernard L, Hedde M, Pelosi C, Villenave C, Chenu C, et al. Fourteen years of evidence for positive effects of conservation agriculture and organic farming on soil life. Agron Sustain Dev. 2014;35(1):169–81. doi: 10.1007/s13593-014-0215-8

[64] van der Laat R, Owen MDK, Liebman M, Leon RG. Postdispersal weed seed predation and invertebrate activity density in three tillage regimes. Weed sci. 2015;63(4):828–38. doi: 10.1614/ws-d-15-00030.1

[65] Wan N-F, Ji X-Y, Kiær LP, Liu S-S, Deng J-Y, Jiang J-X, et al. Ground cover increases spatial aggregation and association of insect herbivores and their predators in an agricultural landscape. Landscape Ecol. 2018;33(5):799–809. doi: 10.1007/s10980-018-0635-y

[66] Brust GE, Stinner BR, McCartney DA. Predator activity and predation in corn agroecosystems. Environmental Entomology. 1986;15(5):1017–21. doi: 10.1093/ee/15.5.1017

[67] Russell MC, Lambrinos J, Records E, Ellen G. Seasonal shifts in ground beetle (Coleoptera: Carabidae) species and functional composition maintain prey consumption in Western Oregon agricultural landscapes. Biological Control. 2017;106:54–63. doi: 10.1016/j.biocontrol.2016.12.008

[68] Logan JD, Wolesensky W, Joern A. Temperature-dependent phenology and predation in arthropod systems. Ecological Modelling. 2006;196(3–4):471–82. doi: 10.1016/j.ecolmodel.2006.02.034

[69] Harwood JD, Phillips SW, Lello J, Sunderland KD, Glen DM, Bruford MW, et al. Invertebrate biodiversity affects predator fitness and hence potential to control pests in crops. Biological Control. 2009;51(3):499–506. doi: 10.1016/j.biocontrol.2009.09.007

[70] Rosenheim JA, Kaya HK, Ehler LE, Marois JJ, Jaffee BA. Intraguild predation among biological-control agents: theory and evidence. Biological Control. 1995;5(3):303–35. doi: 10.1006/bcon.1995.1038

[71] Halpern SL, Bednar D, Chisholm A, Underwood N. Plant‐mediated effects of host plant density on a specialist herbivore of Solanum carolinense. Ecological Entomology. 2013;39(2):217–25. doi: 10.1111/een.12088

[72] Murray PJ, Gregory PJ, Granger SJ, Headon DM, Johnson SN. Dispersal of soil-dwelling clover root weevil (*Sitona lepidus* Gyllenhal, Coleoptera: Curculionidae) larvae in mixed plant communities. Applied Soil Ecology. 2010;46(3):422–5. doi: 10.1016/j.apsoil.2010.09.008

[73] Denys C, Tscharntke T. Plant-insect communities and predator-prey ratios in field margin strips, adjacent crop fields, and fallows. Oecologia. 2002;130(2):315–24. doi: 10.1007/s004420100796 28547156

[74] Andow DA, Ostlie KR. First-Generation European Corn Borer (Lepidoptera: Pyralidae) Response to Three Conservation Tillage Systems in Minnesota. J Economic Entomology. 1990;83(6):2455–61. doi: 10.1093/jee/83.6.2455

[75] Youngman RR, Day ER. European corn borer. Available from: http://www.ext.vt.edu. 2009.

[76] Tooker J. Scout for Western Bean Cutworm in Corn Fields. Available from: https://extension.psu.edu/scout-for-western-bean-cutworm. 2023.

[77] Mihelič R, Pintarič S, Eler K, Suhadolc M. Effects of transitioning from conventional to organic farming on soil organic carbon and microbial community: a comparison of long-term non-inversion minimum tillage and conventional tillage. Biol Fertil Soils. 2024;60(3):341–55. doi: 10.1007/s00374-024-01796-y

[78] Puech C, Baudry J, Joannon A, Poggi S, Aviron S. Organic vs. conventional farming dichotomy: Does it make sense for natural enemies? Agriculture, Ecosystems & Environment. 2014;194:48–57. doi: 10.1016/j.agee.2014.05.002

[79] Reddersen J. The arthropod fauna of organic versus conventional cereal fields in Denmark. Biological Agriculture & Horticulture. 1997;15(1–4):61–71. doi: 10.1080/01448765.1997.9755182

[80] Jeffries DL, Chapman J, Roy HE, Humphries S, Harrington R, Brown PMJ, et al. Characteristics and drivers of high-altitude ladybird flight: insights from vertical-looking entomological radar. PLoS One. 2013;8(12):e82278. doi: 10.1371/journal.pone.0082278 24367512 PMC3867359

[81] Sorribas J, González S, Domínguez-Gento A, Vercher R. Abundance, movements and biodiversity of flying predatory insects in crop and non-crop agroecosystems. Agron Sustain Dev. 2016;36(2). doi: 10.1007/s13593-016-0360-3

[82] Pons X, Núñez E, Lumbierres B, Albajes R. Epigeal aphidophagous predators and the role of alfalfa as a reservoir of aphid predators for arable crops. Eur J Entomol. 2005;102(3):519–25. doi: 10.14411/eje.2005.074

[83] Rand TA, Tylianakis JM, Tscharntke T. Spillover edge effects: the dispersal of agriculturally subsidized insect natural enemies into adjacent natural habitats. Ecol Lett. 2006;9(5):603–14. doi: 10.1111/j.1461-0248.2006.00911.x 16643305

[84] Champagne RJ, Wallace JM, Curran WS, Barbercheck ME. Rotational no‐till and tillage‐based organic corn produce management tradeoffs in the Northeast. Agronomy J. 2021;113(6):5348–61. doi: 10.1002/agj2.20823

[85] Champagne RJ, Wallace JM, Curran WS, Baraibar B. Agronomic and economic tradeoffs between alternative cover crop and organic soybean sequences. Renew Agric Food Syst. 2019;36(1):17–25. doi: 10.1017/s1742170519000437

[86] Jernigan AB, Wickings K, Mohler CL, Caldwell BA, Pelzer CJ, Wayman S, et al. Legacy effects of contrasting organic grain cropping systems on soil health indicators, soil invertebrates, weeds, and crop yield. Agricultural Systems. 2020;177:102719. doi: 10.1016/j.agsy.2019.102719

[87] Pennsylvania Department of Agriculture. The organic agriculture industry in Pennsylvania economic impact, market dynamics, and opportunities for growth. 2024. Available from: https://www.pa.gov/content/dam/copapwp-pagov/en/pda/documents/business-industry/papreferredorganic/documents/PA%20Organic%20Agriculture.pdf

[88] United States Department of Agriculture. National organic grain and feedstuffs report. 2024. Available from: https://www.ams.usda.gov/market-news/organic

[89] Jabbour R, Pisani-Gareau T, Smith RG, Mullen C, Barbercheck M. Cover crop and tillage intensities alter ground-dwelling arthropod communities during the transition to organic production. Renew Agric Food Syst. 2015;31(4):361–74. doi: 10.1017/s1742170515000290

[90] Boomsma CR, Santini JB, West TD, Brewer JC, McIntyre LM, Vyn TJ. Maize grain yield responses to plant height variability resulting from crop rotation and tillage system in a long-term experiment. Soil and Tillage Research. 2010;106(2):227–40. doi: 10.1016/j.still.2009.12.006

