# Supplementary material for: Effect of tillage system on epigeal and foliar insect predation in an organic cropping system in Pennsylvania, USA
Source: PLoS One. 2025 Jul 31;20(7):e0328896. doi: 10.1371/journal.pone.0328896 (PMC12312884; doi:10.1371/journal.pone.0328896)
Supplement: S1 Table — (DOCX) [file pone.0328896.s001.docx]

**Supplementary Materials**

**S 1 Table**. Management strategies for four organic feed grain and forage systems showing the cropping sequence for Entry 3 (Wheat-Corn-Soybean) (after Tillotson et al. 2025)

|  |  | **System 1** | **System 2** | **System 3** | **System 4** |
| --- | --- | --- | --- | --- | --- |
| **Management strategy** | | Cash grain crops managed with inversion tillage, integrating winter cover crops using reduced tillage methods | Soybean phase of rotation managed with shallow tillage using a HSD, intended to reduce the intensity and depth of soil disturbance | Reduced tillage system intended to reduce disturbance to the extent possible | Perennial forage legume-grass mixture as a minimal soil disturbance baseline |
| **Year 1 (2021)** | **Winter** | Wheat planted using chisel tillage | Wheat planted with HSD | Wheat planted with HSD | Oat residue |
|  | **Spring** | Medium red clover no-till drilled into wheat | Wheat | Medium red clover no-till drilled into wheat | Alfalfa/orchardgrass mix planted with chisel plow |
|  | **Summer** | Wheat harvested | Wheat harvested | Red clover | Alfalfa/orchardgrass mix |
|  | **Fall** | Cereal rye no-till drilled into red clover | Oat/Austrian winter pea/forage radish cover crop mixture planted with HSD | Cereal rye no-till drilled into red clover | Alfalfa/orchardgrass mix |
| **Year 2 (2022)** | **Winter** | Cereal rye | Oat/Austrian winter pea/forage radish mixture | Cereal rye/red clover | Alfalfa/orchardgrass mix |
|  | **Spring** | Cereal rye/red clover terminated by inversion tillage | Cover crop terminated with HSD | Cereal rye/red clover terminated by inversion tillage | Alfalfa/orchardgrass mix |
|  | **Summer** | Corn planted Cover crop mixture interseeded into corn | Corn planted | Corn planted Cover crop mixture interseeded into corn | Alfalfa/orchardgrass mix |
|  | **Fall** | Corn harvested | Corn harvested Cereal rye planted with HSD | Corn harvested Cereal rye planted with HSD | Alfalfa/orchardgrass mix |
| **Year 3 (2023)** | **Winter** | Cover crop mixture | Cereal rye | Cereal rye | Alfalfa/orchardgrass mix |
|  | **Spring** | Cover crop mixture terminated with inversion tillage | Cereal rye terminated with HSD | Cereal rye terminated with roller crimper | Alfalfa/orchardgrass mix |
|  | **Summer** | Soybean planted | Soybean planted | Soybean no-till planted | Alfalfa/orchardgrass mix |
|  | **Fall** | Soybean harvested | Soybean harvested | Soybean harvested | Alfalfa/orchardgrass mix |
