# Supplementary material for: Effect of tillage system on epigeal and foliar insect predation in an organic cropping system in Pennsylvania, USA
Source: PLoS One. 2025 Jul 31;20(7):e0328896. doi: 10.1371/journal.pone.0328896 (PMC12312884; doi:10.1371/journal.pone.0328896)
Supplement: S2 Table — (DOCX) [file pone.0328896.s002.docx]

**Supplementary Materials**

**S 2 Table.** Field operations by experimental system, rotation entry point, and date (after Tillotson et al. 2025)

| **Date** | **Entry** | **System 1** | **System 2** | **System 3** | **System 4** | **Operation** |
| --- | --- | --- | --- | --- | --- | --- |
| 17-Mar-2021 | 2 | x |  | x |  | frost seed red clover |
| 5-Apr-2021 | 1 | x | x | x | x | mow corn stubble |
| 5-Apr-2021 | 3 |  |  |  | x | chisel plow, disc x 2, s-tine harrow |
| 7-Apr-2021 | 3 |  |  |  | x | plant alfalfa, cultimulch |
| 21-Apr-2021 | 1 |  | x |  |  | mow cereal rye pre soybean |
| 23-Apr-2021 | 1 |  | x |  |  | HSD 1x time, 2-3 inches |
| 27-Apr-2021 | 3 | x |  | x |  | mow red clover |
| 27-Apr-2021 | 1 | x |  |  | x | mow interseeded cover crop |
| 27-Apr-2021 | 3 | x |  | x |  | moldboard plow red clover pre corn |
| 28-Apr-2021 | 1 | x |  |  | x | moldboard plow interseeded cover crop |
| 3-May-2021 | 1 |  | x |  |  | HSD cereal rye stubble |
| 6-May-2021 | 1 | x |  |  | x | disc pre soybean |
| 6-May-2021 | 3 | x | x | x |  | disc pre corn |
| 6-May-2021 | 3 |  | x |  |  | mow cover crops, HSD pre corn |
| 12-May-2021 | 3 | x | x | x |  | spread poultry litter |
| 14-May-2021 | 3 | x | x | x |  | s-tine corn |
| 18-May-2021 | 3 | x | x | x |  | cultimulch corn |
| 18-May-2021 | 1 | x | x |  | x | s-tine, cultimulch soybean |
| 19-May-2021 | 3 | x | x | x |  | plant corn, 35k/ac, 2.5'' depth |
| 24-May-2021 | 3 | x | x | x |  | tine weed corn |
| 25-May-2021 | 1 |  |  | x |  | roller crimp, plant no-till soybean |
| 27-May-2021 | 1 | x | x |  | x | plant, cultimulch tilled soybean |
| 27-May-2021 | 3 | x | x | x |  | tine weed corn |
| 2-Jun-2021 | 1 | x | x |  | x | tine weed soybean |
| 7-Jun-2021 | 3 | x | x | x |  | cultimulched previous corn |
| 7-Jun-2021 | 3 | x | x | x |  | replant corn |
| 7-Jun-2021 | 3 | x | x | x |  | s-tine to kill previous corn |
| 8-Jun-2021 | 1 | x | x |  | x | tine weed beans, x 2 |
| 10-Jun-2021 | 3 | x | x | x |  | tine weed corn |
| 18-Jun-2021 | 1 | x | x |  | x | cultivate tilled beans |
| **23-Jun-2021** | **1,3** | **x** | **x** | **x** | **x** | **sentinel soil surface predation** |
| 24-Jun-2021 | 3 | x | x | x |  | cultivate corn |
| 24-Jun-2021 | 1 | x | x |  | x | cultivate tilled beans |
| 28-Jun-2021 | 1 |  |  | x |  | high residue cultivate no-till beans |
| 30-Jun-2021 | 3 |  |  |  | x | mow, rake alfalfa |
| 2-Jul-2021 | 3 |  |  |  | x | harvest alfalfa |
| 7-Jul-2021 | 3 | x | x | x |  | cultivate corn |
| 7-Jul-2021 | 3 | x |  |  |  | interseed 20, 8, 1 lb/ac ryegrass, crimson clover, radish |
| 21-Jul-2021 | 1 |  |  | x |  | high residue cultivate no-till beans, x 2 |
| 21-Jul-2021 | 1 | x | x |  | x | cultivate tilled soybeans, x 2 |
| 23-Jul-2021 | 2 | x | x | x | x | harvest wheat |
| 13-Aug-2021 | 3 |  |  |  | x | mow, rake alfalfa |
| 16-Aug-2021 | 2 |  | x |  | x | mow post wheat |
| 24-Aug-2021 | 2 |  |  |  | x | chisel plow, cultimulch, disc, s-tine post wheat |
| 25-Aug-2021 | 2 |  | x |  |  | HSD post wheat |
| 7-Sep-2021 | 2 |  | x |  | x | cultimulch post wheat |
| 8-Sep-2021 | 2 |  | x |  |  | plant oat/pea/radish mix |
| 8-Sep-2021 | 2 |  |  |  | x | plant oats |
| 20-Sep-2021 | 2 | x |  | x |  | harvest red clover |
| **27-Sep-2021** | **2,3** | **x** | **x** | **x** | **x** | **sentinel soil surface predation** |
| 7-Oct-2021 | 3 | x | x | x |  | harvest corn |
| 8-Oct-2021 | 3 | x | x | x |  | stalk chop corn residue |
| 11-Oct-2021 | 3 |  | x | x |  | HSD after corn, 6-8'‘ depth |
| 11-Oct-2021 | 3 |  | x | x |  | HSD after corn 2x, 4-5'' depth |
| 13-Oct-2021 | 3 |  | x | x |  | plant cereal rye |
| 14-Oct-2021 | 2 | x |  | x |  | drill cereal rye into clover, 60lb/ac |
| 20-Oct-2021 | 1 | x | x | x | x | plot combine |
| 22-Oct-2021 | 1 | x |  |  | x | chisel plow |
| 22-Oct-2021 | 1 | x | x | x | x | cultimulch |
| 22-Oct-2021 | 1 |  | x | x |  | HSD |
| 22-Oct-2021 | 1 | x | x | x | x | apply manure, s-tine |
| 28-Oct-2021 | 1 | x | x | x | x | plant wheat |
| 3-Nov-2021 | 3 |  |  |  | x | mow, rake alfalfa |

| **Date** | **Entry** | **System 1** | **System 2** | **System 3** | **System 4** | **Operation** |
| --- | --- | --- | --- | --- | --- | --- |
| 3-Mar-2022 | 1 | x |  | x |  | frost seed red clover |
| 13-Apr-2022 | 2 |  |  |  | x | chisel plow, cultimulch, disc, s-tine, brillion seed alfalfa |
| 25-Apr-2022 | 2 | x |  | x |  | mow clover/rye pre corn |
| 28-Apr-2022 | 2 | x | x | x |  | apply poultry litter pre corn |
| 29-Apr-2022 | 3 |  | x |  |  | HSD, 3'', pre soybean |
| 29-Apr-2022 | 3 | x | x |  |  | mow cereal rye and interseed cover crop pre soybean |
| 29-Apr-2022 | 2 | x |  | x |  | moldboard plow 9-10'' depth, pre corn |
| 29-Apr-2022 | 3 | x |  |  |  | moldboard plow 9-10'' depth, pre soybean |
| 10-May-2022 | 2 |  | x |  |  | HSD |
| 12-May-2022 | 2 | x | x | x |  | disc pre corn |
| 12-May-2022 | 3 | x | x |  |  | disc pre soy |
| 12-May-2022 | 2 | x | x | x |  | s-tine |
| 12-May-2022 | 3 | x | x |  |  | s-tine |
| 13-May-2022 | 2 | x | x | x |  | cultimulch |
| 13-May-2022 | 3 | x | x |  |  | cultimulch |
| 24-May-2022 | 3 |  |  |  | x | harvest alfalfa |
| 25-May-2022 | 3 |  |  | x |  | plant no-till soybean |
| 25-May-2022 | 3 |  |  |  | x | bale alfalfa |
| 25-May-2022 | 3 |  |  | x |  | roller crimp no-till soybean |
| 31-May-2022 | 3 | x | x |  |  | plant soybean |
| 31-May-2022 | 2 | x | x | x |  | plant corn |
| 31-May-2022 | 3 | x | x |  |  | cultimulch |
| 31-May-2022 | 2 | x | x | x |  | cultimulch |
| 3-Jun-2022 | 3 | x | x |  |  | tine weed soybean |
| 3-Jun-2022 | 2 | x | x | x |  | tine weed corn |
| 10-Jun-2022 | 2 | x | x | x |  | rotary hoe corn |
| 10-Jun-2022 | 3 | x | x |  |  | rotary hoe soybean |
| 16-Jun-2022 | 2 | x | x | x |  | rotary hoe corn |
| 16-Jun-2022 | 3 | x | x |  |  | rotary hoe soybean |
| 20-Jun-2022 | 2 | x | x | x |  | cultivate corn |
| 20-Jun-2022 | 3 | x | x |  |  | cultivate soybean |
| **24-June-2022** | **2,3** | **x** | **x** | **x** | **x** | **sentinel soil surface predation** |
| 28-Jun-2022 | 3 | x | x |  |  | cultivated soybean |
| 28-Jun-2022 | 2 | x | x | x |  | cultivated corn |
| 5-Jul-2022 | 2 | x |  |  |  | interseed ryegrass, crimson clover, radish |
| 5-Jul-2022 | 3 |  |  | x |  | high residue cultivate no-till soybean |
| 8-Jul-2022 | 2, 3 |  |  |  | x | harvest alfalfa |
| 8-Jul-2022 | 2,3 |  |  |  | x | rake 1- and 2-year alfalfa |
| 8-Jul-2022 | 3 | x | x |  |  | cultivate soybean |
| 11-Jul-2022 | 2,3 |  |  |  | x | rake alfalfa |
| 13-Jul-2022 | 3 |  |  | x |  | high residue cultivate no-till soybean |
| 20-Jul-2022 | 1 | x | x | x | x | harvest wheat |
| 11-Aug-2022 | 3 |  |  |  | x | harvest 2-year alfalfa |
| 16-Aug-2022 | 3 |  |  |  | x | rake, bale 2-year alfalfa |
| 16-Aug-2022 | 1 |  | x |  | x | mowed wheat stubble |
| 17-Aug-2022 | 2 |  |  |  | x | mow weeds above alfalfa |
| 17-Aug-2022 | 1 |  |  |  | x | chisel plow, cultimulch, disc post wheat |
| 17-Aug-2022 | 1 |  | x |  |  | HSD post wheat |
| 17-Aug-2022 | 1 |  |  |  | x | s-tine |
| 10-Sep-2022 | 1 |  | x |  | x | cultimulch |
| 10-Sep-2022 | 1 |  | x |  |  | plant oat/pea/radish cover crop |
| 10-Sep-2022 | 1 |  |  |  | x | plant oats |
| 13-Sep-2022 | 1 | x |  | x |  | mow clover |
| 13-Sep-2022 | 2 |  |  |  | x | mow alfalfa |
| 16-Sep-2022 | 1 | x |  | x |  | bale clover |
| 16-Sep-2022 | 2 |  |  |  | x | bale alfalfa |
| **27-Sep-2022** | **2,3** | **x** | **x** | **x** | **x** | **sentinel soil surface predation** |
| 6-Oct-2022 | 3 |  |  |  | x | rake, bale alfalfa |
| 7-Oct-2022 | 2 | x | x | x |  | harvest corn |
| 12-Oct-2022 | 2 |  |  | x |  | seed cereal rye post corn, 3 bu/ac |
| 12-Oct-2022 | 2 |  | x |  |  | seed cereal rye post corn, 3 bu/ac |
| 12-Oct-2022 | 1 | x |  | x |  | seed cereal rye into clover |
| 17-Oct-2022 | 3 | x | x | x |  | harvest soybeans, apply manure |
| 18-Oct-2022 | 3 | x |  |  |  | chisel plow, cultimulch, disc post soybean |
| 18-Oct-2022 | 3 |  | x | x |  | cultimulch, HSD post soybean |
| 18-Oct-2022 | 3 | x | x | x |  | s-tine, plant wheat |

| **Date** | **Entry** | **System 1** | **System 2** | **System 3** | **System 4** | **Operation** |
| --- | --- | --- | --- | --- | --- | --- |
| 21-Feb-2023 | 3 | x |  | x |  | frost seeded medium red clover |
| 4-Apr-2023 | 1 |  |  |  | x | chisel plow, heavy disc, s-tine, cultimulch |
| 10-Apr-2023 | 1 |  |  |  | x | plant, cultimulch alfalfa |
| 10-May-2023 | 1 | x | x | x |  | mow cc |
| 10-May-2023 | 2 | x | x |  |  | mow cc |
| 10-May-2023 | 1 | x |  | x |  | moldboard plow |
| 10-May-2023 | 2 | x |  |  |  | moldboard plow |
| 11-May-2023 | 1 |  | x |  |  | high speed disc 2x |
| 11-May-2023 | 2 |  | x |  |  | high speed disc 2x |
| 11-May-2023 | 1 | x | x | x |  | heavy disc |
| 11-May-2023 | 2 | x | x |  |  | heavy disc |
| 11-May-2023 | 1 | x | x | x |  | s-tine |
| 11-May-2023 | 2 | x | x |  |  | s-tine |
| 12-May-2023 | 1 | x | x | x |  | cultimulch |
| 12-May-2023 | 2 | x | x |  |  | cultimulch |
| 15-May-2023 | 1 | x | x | x |  | apply manure |
| 24-May-2023 | 2 |  |  | x |  | roller crimp, plant no-till soybeans |
| 25-May-2023 | 2,3 |  |  |  | x | mow, rake alfalfa off plot |
| 7-Jun-2023 | 1 | x | x | x |  | cultimulch, plant corn |
| 7-Jun-2023 | 2 | x | x |  |  | cultimulch, plant soybean |
| 10-Jun-2023 | 1 | x | x | x |  | tine weed |
| 10-Jun-2023 | 2 | x | x |  |  | tine weed |
| **15-Jun-2023** |  |  |  |  |  | **start western bean cutworm pheromone trapping** |
| 18-Jun-2023 | 1 | x | x | x |  | rotary hoe |
| 18-Jun-2023 | 1,2 |  | x |  |  | cultivate |
| 20-Jun-2023 | 1 | x | x | x |  | cultivate |
| 20-Jun-2023 | 2 | x | x |  |  | rotary hoe, cultivate |
| **29-Jun-2023** | **1,2** | **x** | **x** | **x** | **x** | **sentinel soil surface predation** |
| 30-Jun-2023 | 1 | x | x | x |  | cultivate corn |
| 30-Jun-2023 | 2 | x | x |  |  | cultivate soybean |
| 6-Jul-2023 | 1 | x | x | x |  | cultivate corn |
| 6-Jul-2023 | 2 | x | x |  |  | cultivate soybean |
| 6-Jul-2023 | 1,2,3 |  |  |  | x | alfalfa yield strip |
| 6-Jul-2023 | 1,2,3 |  |  |  | x | mow alfalfa |
| 10-Jul-2023 | 1,2,3 |  |  |  | x | bale alfalfa |
| **10-Jul-2023** | **1** | **x** | **x** | **x** |  | **start timed predator counts** |
| 11-Jul-2023 | 2 |  |  | x |  | high residue cultivate no-till beans |
| **12-Jul-2023** | **1** | **x** | **x** | **x** |  | **start European corn borer sentinel egg observation** |
| 13-Jul-2023 | 1 | x | x | x |  | cultivate corn |
| 13-Jul-2023 | 2 | x | x |  |  | cultivate soybean |
| 19-Jul-2023 | 1 | x |  |  |  | interseeded |
| 19-Jul-2023 | 2 | x | x |  |  | cultivate soybean |
| **24-July-2023** | **1** | **x** | **x** | **x** |  | **start western bean cutworm egg observations** |
| 26-Jul-2023 | 3 | x | x | x |  | harvested wheat |
| 1-Aug-2023 | 2 |  |  | x |  | high residue cultivate |
| 17-Aug-2023 | 1,2,3 |  |  |  | x | alfalfa yield strip |
| 17-Aug-2023 | 1,2,3 |  |  |  | x | mow alfalfa |
| 17-Aug-2023 | 3 |  | x |  |  | mow weeds |
| 17-Aug-2023 | 3 | x |  | x |  | mow weeds above clover |
| 22-Aug-2023 | 1,2,3 |  |  |  | x | rake alfalfa off plot |
| 5-Sep-2023 | 3 |  | x |  |  | HSD, cultimulch |
| 8-Sep-2023 | 3 |  | x |  |  | cultimulch |
| 8-Sep-2023 | 3 |  | x |  |  | plant oat/pea/radish |
| 12-Sep-2023 | 3 | x |  | x |  | mow weeds above clover |
| **14-Sep-2023** | **1,2** | **x** | **x** | **x** | **x** | **sentinel soil surface predation** |
| 29-Sep-2023 | 1,2,3 |  |  |  | x | mow alfalfa |
| **8-Aug-2023** | **1** | **x** | **x** | **x** |  | **end western bean cutworm egg observations** |
| **8-Aug-2023** | **1** | **x** | **x** | **x** |  | **end timed predator counts** |
| **9-Aug-2023** | **1** | **x** | **x** | **x** |  | **end European corn borer egg observations** |
| **28-Aug-2023** |  |  |  |  |  | **end western bean cutworm pheromone trapping** |
| **28-Aug-2023** | **1** | **x** | **x** | **x** |  | **start caterpillar collection** |
| **28-Aug-2023** | **1** | **x** | **x** | **x** |  | **start corn ear damage observations** |
| **30-Aug-2023** | **1** | **x** | **x** | **x** |  | **end caterpillar collection** |
| **30-Aug-2023** | **1** | **x** | **x** | **x** |  | **end corn ear damage observations** |
| 4-Oct-2023 | 1,2,3 |  |  |  | x | raked alfalfa off plot |
| 12-Oct-2023 | 3 | x |  | x |  | drillcereal rye into clover |
| 25-Oct-2023 | 1 | x | x | x |  | harvest corn |
| 25-Oct-2023 | 1 |  | x | x |  | mow corn stalks |
| 26-Oct-2023 | 1 |  | x | x |  | HSD x2, drill 3 bu/ac cereal rye, |
| 6-Nov-2023 | 2 | x | x | x |  | harvested soybean |
| 6-Nov-2023 | 2 | x |  |  |  | chisel plow, disc, cultimulch |
| 7-Nov-2023 | 2 |  | x | x |  | HSD |
| 7-Nov-2023 | 2 | x | x | x |  | plant wheat |
