# Supplementary material for: Effect of tillage system on epigeal and foliar insect predation in an organic cropping system in Pennsylvania, USA
Source: PLoS One. 2025 Jul 31;20(7):e0328896. doi: 10.1371/journal.pone.0328896 (PMC12312884; doi:10.1371/journal.pone.0328896)
Supplement: S3 Table — (DOCX) [file pone.0328896.s003.docx]

**Supplementary Materials**

**S 3 Table.** Annual and accumulated frequency and estimated soil disturbance ratings (SDR) for experimental rotation entries and cropping systems.

|  |  |  | Annual Number of Disturbances | | | | Annual SDR | | | | Rotation Number of Disturbances | | | | Rotation SDR | | | |
| --- | --- | --- | --- | --- | --- | --- | --- | --- | --- | --- | --- | --- | --- | --- | --- | --- | --- | --- |
| Entry | Year | Crop | Sys  1 | Sys  2 | Sys  3 | Sys  4 | Sys  1 | Sys  2 | Sys  3 | Sys  4 | Sys  1 | Sys  2 | Sys  3 | Sys  4 | Sys  1 | Sys  2 | Sys  3 | Sys  4 |
| 1 | 2021 | Soy | 21 | 21 | 13 | 21 | 304 | 310 | 160 | 304 | 38 | 39 | 32 | 37 | 525 | 574 | 429 | 520 |
|  | 2022 | Wheat | 7 | 6 | 7 | 9 | 38 | 74 | 38 | 117 | 45 | 45 | 39 | 46 | 563 | 648 | 467 | 637 |
|  | 2023 | Corn | 16 | 21 | 19 | - | 240 | 330 | 291 | - | 61 | 66 | 58 | - | 803 | 978 | 758 | - |
|  |  | Alfalfa | - | - | - | 15 | - | - | - | 124 | - | - | - | 61 | - | - | - | 761 |
| Entry 1 Rotation Disturbance | | | 44 | 48 | 39 | 45 | 582 | 714 | 489 | 545 | 61 | 66 | 58 | 61 | 803 | 978 | 758 | 761 |
| 2 | 2021 | Wheat | 4 | 6 | 4 | 9 | 29 | 74 | 29 | 107 | 25 | 26 | 24 | 30 | 303 | 340 | 294 | 382 |
|  | 2022 | Corn | 15 | 15 | 16 | - | 205 | 226 | 229 | - | 19 | 21 | 20 | - | 508 | 566 | 523 | - |
|  |  | Alfalfa | - | - | - | 13 | - | - | - | 93 | - | - | - | 22 | - | - | - | 475 |
|  | 2023 | Soy | 19 | 18 | 7 | - | 311 | 318 | 83 | - | 38 | 40 | 26 | - | 819 | 884 | 606 | - |
|  |  | Alfalfa | - | - | - | 12 | - | - | - | 36 | - | - | - | 33 | - | - | - | 511 |
| Entry 2 Rotation Disturbance | | | 38 | 39 | 27 | 21-22 | 545 | 618 | 341 | 143-200 | 38 | 40 | 26 | 52-63 | 819 | 884 | 606 | 382-511 |
| 3 | 2021 | Corn | 19 | 21 | 21 | - | 231 | 274 | 279 | - | 25 | 29 | 27 | - | 272 | 380 | 320 | - |
|  |  | Alfalfa | - | - | - | 14 | - | - | - | 126 | - | - | - | 21 | - | - | - | 232 |
|  | 2022 | Soy | 20 | 19 | 10 | - | 292 | 279 | 123 | - | 45 | 48 | 37 | - | 564 | 654 | 443 | - |
|  |  | Alfalfa | - | - | - | 13 | - | - | - | 39 | - | - | - | 34 | - | - | - | 271 |
|  | 2023 | Wheat | 6 | 6 | 6 | - | 31 | 88 | 31 | - | 51 | 54 | 43 | - | 595 | 742 | 474 | - |
|  |  | Alfalfa | - | - | - | 13 | - | - | - | 39 | - | - | - | 47 | - | - | - | 310 |
| Entry 3 Rotation Disturbance | | | 45 | 46 | 37 | 13-14 | 554 | 641 | 433 | 39-126 | 51 | 54 | 43 | 21-47 | 595 | 742 | 474 | 232-310 |

**S 4 Table.** Schedule of activities for predation on eggs of Western bean cutworm (WBC) and European corn borer (ECB) on corn foliage in 2023. GDD= Growing Degree Days, Rock Springs, PA, using the NEWS Cornell GDD calculator (https://newa.cornell.edu/degree-day-calculator/).

| **Field operation or assay** | **Date or date range** | **Corn GDD** | **WBC GDD** | **WBC estimated emergence** |
| --- | --- | --- | --- | --- |
| Planted corn | 17 June | 9 | 469 | <25% |
| WBC pheromone trapping | 15 June – 28 August | 102 - 1616 | 441 – 1955 | <25% to 75% |
| WBC egg predation assessments | 24 July – 8 August | 897 - 1213 | 1236 – 1553 | <25% to 75% |
| Timed predator counts | 10 July – 8 August | 589 - 1213 | 928 – 1553 | <25% to 75% |
| ECB sentinel predation | 12 July – 9 August | 634 - 1236 | 973 – 1575 | <25% to >75% |
| Interseeded System 1 (inversion) corn | 19 July | 796 | 1136 | <25% |
| Caterpillar collection | 28 – 30 August | 1616 - 1650 | 1955 – 1989 | >75% |
| Corn ear damage assessment | 28– 30 August | 1616 - 1650 | 1955 – 1989 | >75% |
| Harvested corn | 25 October | 2196 | 2535 | >75% |

**S 5 Table.** Total number of western bean cutworm (WBC) egg masses, total number of individual WBC eggs observed, and mean percent of hatched and predated WBC egg mass observed in corn in 2023.

| **System** | **Total no. egg masses** | **Total no. eggs** | **Mean % hatch/egg mass** | **Mean % predated/ egg mass** |
| --- | --- | --- | --- | --- |
| 1  (Inversion Till) | 78 | 5226 | 62.6 ± 12.1% | 14.4 ± 21.3% |
| 2  (Shallow Till) | 94 | 6206 | 71.8 ± 14.3% | 18.3 ± 19.4% |
| 3  (Reduced Till) | 60 | 4536 | 72.1 ± 15.9% | 18.4 ± 20.3% |


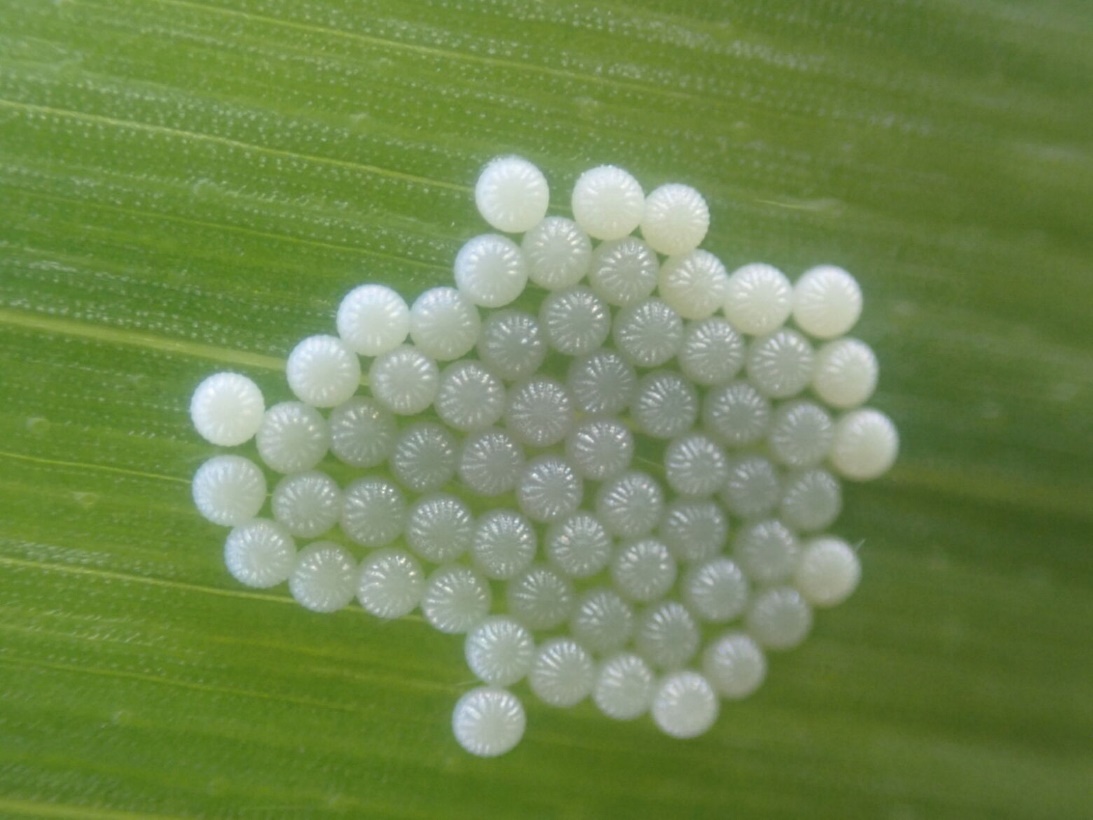


**S1 Fig.:** Freshly oviposited western bean cutworm egg mass on corn leaf. Morphological features include white coloration, round dome shape, and “dumpling-like” indents on dorsal side.


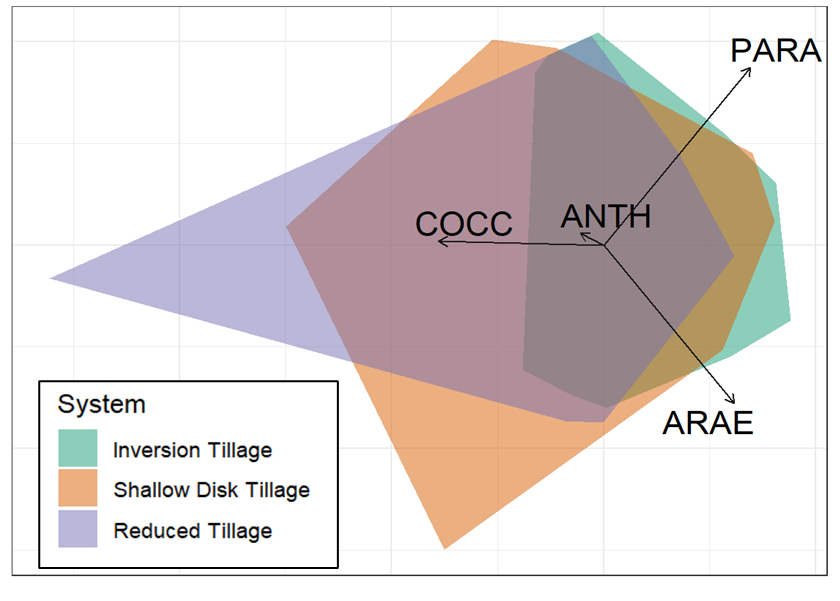


**S Fig. 2.** NMDS plot depicting arthropod predator community composition on corn foliage in 2023. Taxonomic groups that accounted for greater than 1% of all observations including: COCC (Coccinellidae), ANTH (Anthocoridae), ARAE (Aranae), PARA (parasitoid wasps). The green shape represents the inversion tillage system (moldboard plow), the orange shape represents the shallow tillage system (high-speed disk), and the purple shape represented the reduced tillage system (moldboard plow in corn, no-till planting in the previous soybean crop).
