# Supplementary material for: Effect of tillage system on epigeal and foliar insect predation in an organic cropping system in Pennsylvania, USA
Source: PLoS One. 2025 Jul 31;20(7):e0328896. doi: 10.1371/journal.pone.0328896 (PMC12312884; doi:10.1371/journal.pone.0328896)
Supplement: S4 Table — GDD = Growing Degree Days, Rock Springs, PA, using the NEWS Cornell GDD calculator (https://newa.cornell.edu/degree-day-calculator/). (DOCX) [file pone.0328896.s004.docx]

**Supplementary Materials**

**S 4 Table.** Schedule of activities for predation on eggs of Western bean cutworm (WBC) and European corn borer (ECB) on corn foliage in 2023. GDD = Growing Degree Days, Rock Springs, PA, using the NEWS Cornell GDD calculator (https://newa.cornell.edu/degree-day-calculator/).

| **Field operation or assay** | **Date or date range** | **Corn GDD** | **WBC GDD** | **WBC estimated emergence** |
| --- | --- | --- | --- | --- |
| Planted corn | 17 June | 9 | 469 | <25% |
| WBC pheromone trapping | 15 June – 28 August | 102 - 1616 | 441 – 1955 | <25% to 75% |
| WBC egg predation assessments | 24 July – 8 August | 897 - 1213 | 1236 – 1553 | <25% to 75% |
| Timed predator counts | 10 July – 8 August | 589 - 1213 | 928 – 1553 | <25% to 75% |
| ECB sentinel predation | 12 July – 9 August | 634 - 1236 | 973 – 1575 | <25% to >75% |
| Interseeded System 1 (inversion) corn | 19 July | 796 | 1136 | <25% |
| Caterpillar collection | 28 – 30 August | 1616 - 1650 | 1955 – 1989 | >75% |
| Corn ear damage assessment | 28– 30 August | 1616 - 1650 | 1955 – 1989 | >75% |
| Harvested corn | 25 October | 2196 | 2535 | >75% |
