# Supplementary material for: Effect of tillage system on epigeal and foliar insect predation in an organic cropping system in Pennsylvania, USA
Source: PLoS One. 2025 Jul 31;20(7):e0328896. doi: 10.1371/journal.pone.0328896 (PMC12312884; doi:10.1371/journal.pone.0328896)
Supplement: S5 Table — (DOCX) [file pone.0328896.s005.docx]

**Supplementary Materials**

**S 5 Table.** Total number of western bean cutworm (WBC) egg masses, total number of individual WBC eggs observed, and mean percent of hatched and predated WBC egg mass observed in corn in 2023.

| **System** | **Total no. egg masses** | **Total no. eggs** | **Mean % hatch/egg mass** | **Mean % predated/ egg mass** |
| --- | --- | --- | --- | --- |
| 1  (Inversion Till) | 78 | 5226 | 62.6 ± 12.1% | 14.4 ± 21.3% |
| 2  (Shallow Till) | 94 | 6206 | 71.8 ± 14.3% | 18.3 ± 19.4% |
| 3  (Reduced Till) | 60 | 4536 | 72.1 ± 15.9% | 18.4 ± 20.3% |
