# Supplementary material for: Effect of tillage system on epigeal and foliar insect predation in an organic cropping system in Pennsylvania, USA
Source: PLoS One. 2025 Jul 31;20(7):e0328896. doi: 10.1371/journal.pone.0328896 (PMC12312884; doi:10.1371/journal.pone.0328896)
Supplement: S1 Fig — Morphological features include white coloration, round dome shape, and “dumpling-like” indents on dorsal side. (DOCX) [file pone.0328896.s006.docx]

**Supplementary Materials**

**S1 Fig.:** Freshly oviposited western bean cutworm egg mass on corn leaf. Morphological features include white coloration, round dome shape, and “dumpling-like” indents on dorsal side.


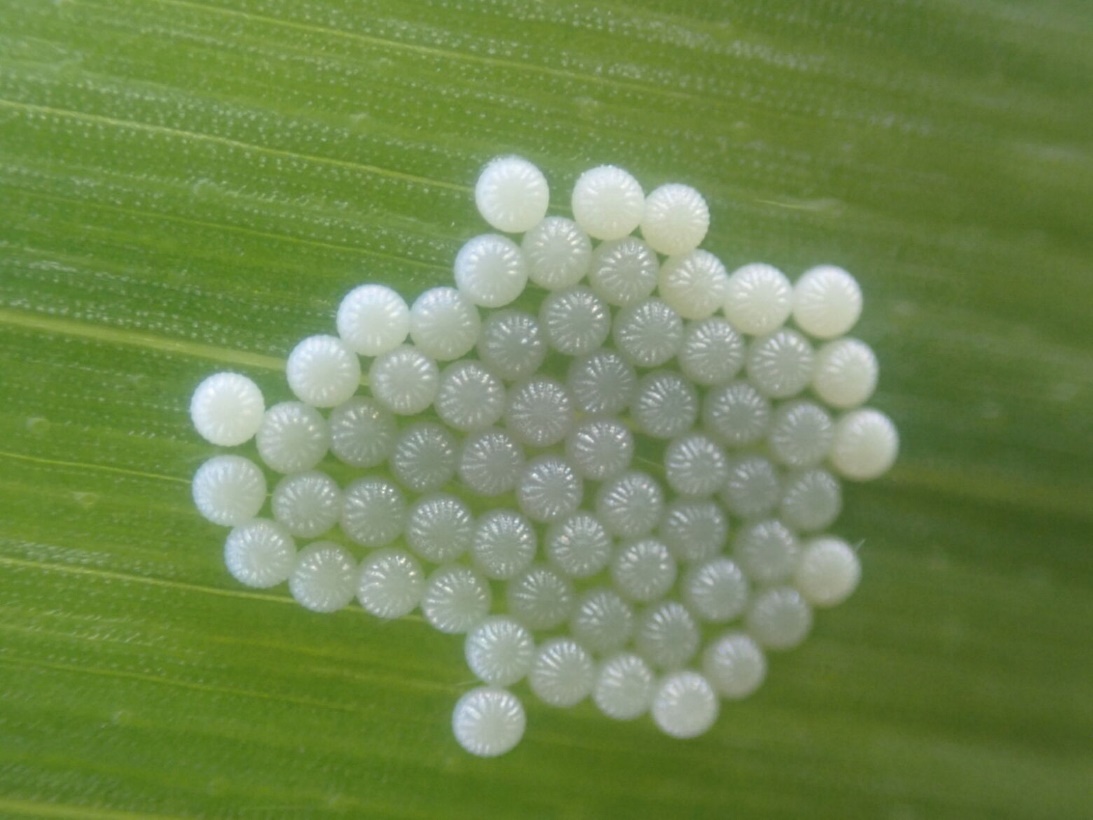


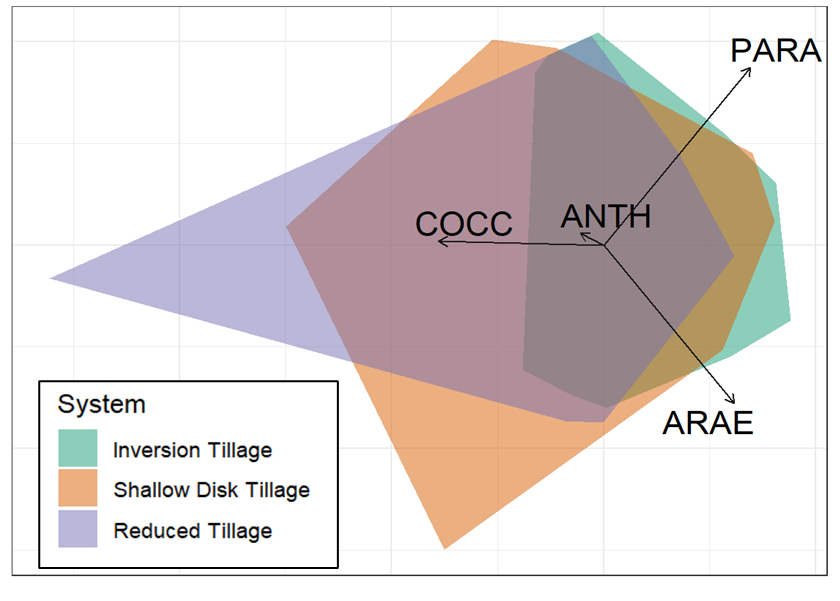


**S Fig. 2.** NMDS plot depicting arthropod predator community composition on corn foliage in 2023. Taxonomic groups that accounted for greater than 1% of all observations including: COCC (Coccinellidae), ANTH (Anthocoridae), ARAE (Aranae), PARA (parasitoid wasps). The green shape represents the inversion tillage system (moldboard plow), the orange shape represents the shallow tillage system (high-speed disk), and the purple shape represented the reduced tillage system (moldboard plow in corn, no-till planting in the previous soybean crop).
